# SSEA3 and CD105 positivity are associated with the treatment potency of human neural crest-derived nasal turbinate stem cells for Alzheimer’s disease

**DOI:** 10.1186/s40035-026-00539-3

**Published:** 2026-03-03

**Authors:** Jung Yeon Lim, Jung Eun Lee, Minho Lee, Haewon Shim, Sang In Park, Soon A. Park, Sin-Soo Jeun, Sheng-Min Wang, Sunghwan Kim, Seung Ho Yang, Hyun Kook Lim, Sung Won Kim

**Affiliations:** 1 Department of Otolaryngology-Head and Neck Surgery, College of Medicine, Seoul St. Mary’s Hospital, The Catholic University of Korea, 222 Banpo-daero Seocho-gu, Seoul, 09591 Republic of Korea; 2 Department of Neurosurgery, College of Medicine, St. Vincent’s Hospital, The Catholic University of Korea, 93 Jungbu-daero, paldal-gu, Suwon-si, Seoul, 16247 Republic of Korea; 3 Department of Life Science, Dongguk University, 30, Pildong-ro 1-gil, Jung-gu, Seoul, 04620 Republic of Korea; 4 Institute of Catholic Integrative Medicine (ICIM), Incheon St. Mary’s Hospital, The Catholic University of Korea, 56 Dongsu-ro, Bupyeong-dong, Bupyeonggu, Incheon, 21431 Republic of Korea; 5 Department of Neurosurgery, Seoul St. Mary’s Hospital, The Catholic University of Korea, 222 Banpo-daero Seocho-gu, Seoul, 09591 Republic of Korea; 6 Department of Psychiatry, Yeouido St. Mary’s Hospital, The Catholic University of Korea, 10 63-ro Yeongdeungpo-gu , Seoul, 07345 Republic of Korea

**Keywords:** Alzheimer’s disease, Cognitive function, Human neural crest-derived nasal turbinate stem cells, Neuroinflammation, SSEA3^+^/CD105^+^ cells, Stem cell therapy

## Abstract

**Background:**

Stem cells have the potential to treat Alzheimer’s disease (AD), but clinical outcomes are unpredictable due to inter-donor differences in stem cell properties. This study aimed to determine whether the pluripotency marker SSEA3 and the mesenchymal marker CD105 positivity are associated with the therapeutic efficacy of human neural crest-derived nasal turbinate stem cells (NTSCs) for AD.

**Methods:**

The therapeutic effects of NTSCs obtained from different donors, with varying percentages of SSEA3^+^/CD105^+^ cells, were explored in 5 × FAD transgenic AD mice and cerebral organoids derived from induced pluripotent stem cells (iPSC) of three AD patients. Neuropathological changes associated with AD were examined, including expression of beta-amyloid, inflammation, and neuronal survival. Cognitive functions were evaluated by the Morris water maze (MWM) test.

**Results:**

NTSCs from different donors improved cognitive function and AD-related neuropathology to varying degrees, depending on the percentage of SSEA3^+^/CD105^+^ cells. Compared with NTSCs with a lower percentage of SSEA3^+^/CD105^+^ cells (NTSCs-L), NTSCs with a higher percentage of SSEA3^+^/CD105^+^ cells (NTSCs-H) showed greater properties in vitro, including proliferative capacity, multilineage differentiation potency, and secretion of neuroprotective cytokines. These properties were comparable to those of pure SSEA3^+^/CD105^+^ cells isolated from NTSCs (NTSCs-SC). Both NTSCs-H and NTSCs-SC improved cognitive function and reduced cerebral Aβ deposition, inflammation, and neuronal death in AD model mice. Furthermore, NTSCs-H and NTSCs-SC decreased Aβ aggregates, tau hyperphosphorylation, neuronal death, microglial numbers, and inflammatory cytokine levels in AD cerebral organoids. However, there was no significant difference in AD-related pathological changes between NTSCs-H and NTSCs-SC treatment groups.

**Conclusions:**

Our findings suggest that SSEA3/CD105 positivity is a potential marker of NTSC therapeutic efficacy for the treatment of AD. Future studies should focus on enhancing the therapeutic potential of SSEA3^+^/CD105^+^ NTSCs by improving their functional efficacy and consistency, and advancing their use in clinical settings.

**Supplementary Information:**

The online version contains supplementary material available at 10.1186/s40035-026-00539-3.

## Background

Alzheimer’s disease (AD) is a progressive neurodegenerative disorder that leads to cognitive decline, interfering activities of daily living. AD is the most common cause of dementia and accounts for 60%–80% of dementia cases [1, 2]. Pathologically, AD is characterized by accumulation of beta-amyloid (Aβ) plaques and tau tangles in the brain, which contribute to the death of nerve cells and progressive loss of brain tissue [3, 4]. There are several medications approved by the FDA to manage the symptoms of AD and slow disease progression [5, 6], but their safety and effectiveness remain a matter of concern.

Stem cells have emerged as a new option to treat or prevent AD [7–10]. Mesenchymal stem cells (MSCs) have the ability to replace damaged neurons and prevent cell loss through secretion of crucial therapeutic molecules [11–13]. Furthermore, they exert significant immunomodulatory effects by producing cytokines, chemokines, and extracellular vesicles that attenuate immune responses, suggesting their potential as immunomodulatory agents [14–16]. Numerous preclinical studies have demonstrated that human MSC transplantation alleviates neuropathology and ameliorates cognitive function in animal models of AD [17, 18].

Despite these promising preclinical findings and the finding that MSCs are safe and nontumorigenic [19], MSC therapy has not shown expected therapeutic effects in clinical trials. Specifically, MSC treatment did not ameliorate neuropathology and cognitive dysfunction in AD patients [20, 21]. Thus, research has focused on identifying MSC sources that yield cells with enhanced therapeutic efficacy in clinical trials. However, identifying suitable types of MSCs for treating AD remains challenging due to the inter-donor variability. The in vitro properties of MSCs derived from different donors varied, as did their in vivo therapeutic efficacy in a mouse model [22–24]. This variability makes it challenging to achieve consistent clinical outcomes across different patients receiving MSC therapy. Developing a strategy to predict the therapeutic efficacy of MSCs and select MSCs with the best therapeutic efficacy will ensure consistent product quality and reproducibility of clinical outcomes for AD.

One type of stem cells with good potential for treating AD is human neural crest-derived nasal turbinate stem cells (NTSCs). NTSCs can be isolated from human inferior nasal turbinate tissue removed during turbinate resection, a minimally invasive procedure that is commonly performed to relieve nasal obstructions resulting from turbinate hypertrophy [25, 26]. Compared with human bone marrow-derived MSCs (BMSCs), NTSCs exhibit greater biological and neurogenic activity in culture, and transplantation of NTSCs has been shown to alleviate neuropathology and cognitive impairment in AD model mice to a greater extent than transplantation of BMSCs [27].

Multilineage differentiating stress-enduring (Muse) cells are adult stem cells double positive for the pluripotency marker SSEA3 and the mesenchymal marker CD105 (SSEA3^+^/CD105^+^); these cells can differentiate into cells of all germ layers in vitro [28–30]. Moreover, unlike embryonic stem cells, Muse cells can be obtained from various adult tissues, including bone marrow and adipose tissue, and are considered nontumorigenic [31]. Muse cells are a subpopulation of MSCs that exert therapeutic effects by regulating the immune system, inhibiting apoptosis, and replacing cells via spontaneous differentiation into specific cell types [29, 31]. These properties make Muse cells potentially valuable for treating neurodegenerative diseases such as AD. Indeed, NTSCs exhibited greater biological and neurogenic property in culture compared with BMSCs, which is linked to a higher percentage of Muse cells present in the NTSC populations [27].

In this study, we investigated the potential of Muse cells (SSEA3^+^/CD105^+^) as a marker for predicting the efficacy of NTSCs in treating AD in pre-clinical settings. NTSCs from five different donors and having different percentages of Muse cells (SSEA3^+^/CD105^+^) were transplanted into 5 × FAD transgenic (Tg) AD model mice. Then Aβ aggregation, inflammation, and neuronal survival in the brain were evaluated. Morris water maze (MWM) test was performed to evaluate the effects of NTSCs on cognitive function. Moreover, the therapeutic effects of NTSCs were compared with Muse cells (SSEA3^+^/CD105^+^) isolated from NTSCs on AD-related pathological changes in both 5 × FAD mice and cerebral organoids (COs) derived from induced pluripotent stem cells (iPSC) of three AD patients (AD-COs).

## Methods

### Ethics approval

All studies were approved by the Institutional Review Board of St. Mary’s Hospital (KC08TISS0341), Yeouido St. Mary’s Hospital (XC22TND10089), and St. Vincent’s Hospital (VC20TIS10005) of the Catholic University of Korea. Written informed consent was obtained from all NTSC donors and from all patients with AD, and the studies followed the Declaration of Helsinki. The participants who donated blood for development of brain organoids had provided written informed consent to participate in this study.

### Isolation and culture of human NTSCs

Experiments utilizing NTSCs were approved by the Institutional Review Board of St. Mary’s Hospital, Catholic University of Korea (KC08TISS0341) and performed in accordance with the Declaration of Helsinki. The participants provided written informed consent to participate in the study prior to surgery. NTSCs were isolated from donated nasal inferior turbinate tissue from human patients who underwent partial turbinectomy as described previously [25, 26]. The tissues were washed with saline and PBS (Thermo Fisher Scientific, Waltham, MA), cut into small pieces, plated on culture dishes, and covered with a sterilized glass coverslip. The glass-covered tissues were incubated in α-minimum essential medium (α-MEM; Thermo Fisher Scientific) supplemented with 1% (*v*/*v*) penicillin/streptomycin (antibiotics, Invitrogen, Carlsbad, CA) and 10% (*v*/*v*) fetal bovine serum (FBS; Thermo Fisher Scientific), in a 37 °C incubator in 5% (*v*/*v*) CO_2_. The tissues were cultured for 3 weeks, with medium change every 2 days. After 3 weeks, the glass coverslips were removed, and cells were harvested from the tissues via 0.25% trypsin in 1 mM EDTA solution. The NTSCs were cultured for 5–6 passages for use in vitro and in vivo.

### Cell transplantation

Male 5 × FAD mice expressing five mutations in human AβPP and PSEN1 (B6SJL-Tg[AβPP *K670N*M671L*I716V*V717I, PSEN1*M146*L286V]6799Vas/J) under the control of the Thy1 promoter (16 weeks of age; Jackson Laboratory, Bar Harbor, ME) were used for cell transplantation. Animal experiments were approved by the Institutional Animal Care and Use Committee of Catholic University and performed in accordance with institutional guidelines (CMCIBC-2023–33-02). The mice were randomly divided into five treatment groups (*n* = 8–10 mice per group): (1) wild-type (WT) C57BL/6 mice treated with PBS (WT-sham group), (2) transgenic (Tg) mice treated with PBS (Tg-sham group), (3) Tg mice treated with NTSCs from five different donors (Tg-NTSC#1–5 groups), (4) Tg mice treated with NTSCs containing the highest percentage of Muse cells (Tg-NTSC-H group), and (5) Tg mice treated with Muse cells isolated from NTSCs (Tg-NTSC-SC group).

The 16-week-old mice were anesthetized with ketamine (50 mg/kg; Zoletil, Virbac Laboratory, Carros, France) and xylazine (10 mg/kg). PBS (3 μL) or suspended NTSCs (1 × 10^5^ cells/3 μL) were slowly injected into the dentate gyrus of the bilateral hippocampus (AP, − 1.94 mm; ML, ± 1.00 mm; DV, − 2.6 mm) at a rate of 0.5 μL/min with a Hamilton syringe with a 26-gauge needle (Hamilton Company, Reno, NV) using a microinjection pump (KD Scientific, Reno, NV) on a stereotaxic device. Antibiotics (gentamicin, 5 mg/kg, s.c.; Shinnpung Pharm, Seoul, Korea) and pain medications (ketoprofen, 5 mg/kg, subcutaneous injection; SDC Pharm, Seoul, Korea) were administered before surgery to prevent infection. After surgery, the surgical site was sterilized, antibiotics (gentamicin, 5 mg/kg, subcutaneous injection; Shinnpung Pharm) and analgesics (ketoprofen, 5 mg/kg, subcutaneous injection; SDC Pharm) were administered once daily for 3–5 days, and the animals were checked at least three times a day. A recovery diet and fluid therapy (normal saline, 0.1 mL/10 g, intraperitoneal injection; Daehan Pharm. Co. Ltd., Seoul, Korea) were provided as needed to help the animals recover.

### Behavioral test

The MWM test was conducted 6 weeks after NTSC transplantation. The apparatus consisted of a circular pool (1.5 m diameter) filled with water (25 °C). Before starting the test, the AD model mice were acclimatized to the test environment by allowing them to freely swim in the pool for 60 s. Then, a platform (10 cm in diameter) was placed 1 cm above the surface of the water, and each mouse was allowed to swim for 60 s, after which they were kept on the platform for 20 s. On the next day, the platform was placed 1 cm below the water surface, and the mice were trained to find the hidden platform in three trials per day for 7–8 days. In the training session, each mouse was placed in a different starting location in one of the quadrants except the platform quadrant, and was allowed to search for the platform for up to 60 s. At the end of each trial, the mouse was dried with a towel and placed in a warm cage to maintain its body temperature. To minimize the experimental variables, the trial was conducted at similar times each day. On day 8 or 9 of the test, a probe trial was conducted to assess memory retention. In the probe trial, the platform was removed from the pool, and each mouse was released into the pool directly opposite to the platform location and allowed to swim freely for 60 s. The mice were automatically monitored, and the time spent in each quadrant (zones 1–4) was recorded via the Smart 3.0 Video Tracking System (Panlab, S.L., Barcelona, Spain).

### Fluorescence immunohistochemistry

Histological analysis was conducted at 7 weeks post-transplantation to evaluate neuropathological changes. The mice were anesthetized with ketamine (50 mg/kg, Zoletil, Virbac Laboratory) and xylazine (10 mg/kg, Rompun, Bayer, Leverkusen, Germany) and then perfused with 4% paraformaldehyde (PFA) (Biosesang, Seongnam, Korea). Brain tissues were collected, fixed, embedded, snap-frozen in liquid nitrogen, and stored at − 80 °C. For immunohistochemistry, brains were cut at 13 μm of thickness using a freezing microtome. To detect Aβ plaques, the sections were pretreated with 97% formic acid for 3 min and then incubated with a mouse anti-Aβ antibody (6E10, 1:100; BioLegend, San Diego, CA, 803002) for 1–2 h at room temperature, followed by incubation with a biotinylated horse anti-mouse IgG antibody (1:200; Vector Laboratories, Burlingame, CA) and FITC-streptavidin (1: 300; Invitrogen^TM^, Carlsbad, CA, 50-112-1547). To detect other target proteins, brain tissue sections were incubated with anti-Iba-1 (1:500; Wako, Osaka, Japan, 019-19741), anti-NeuN (1:200, Merck Millipore, Burlington, MA, ABN78), or anti-human nuclear antigen (HuNu) (1:100, Merck Millipore, MAB1281) antibody followed by Alexa Fluor 488- and Alex Fluor 546-conjugated goat anti-mouse and goat anti-rabbit antibodies (Invitrogen).

To detect apoptotic cells, brain and organoid tissue sections were processed using a TUNEL assay kit (PE-conjugated, Cell Signaling Technology, Danvers, MA, 25879). Nuclei were labeled with DAPI (1:1000, Sigma-Aldrich). Fluorescence was detected using a Zeiss LSM510 confocal microscope (Carl Zeiss, Jena, Germany).

For immunohistochemical staining of COs, cultured COs were fixed with 4% PFA, embedded in paraffin, and sectioned at a thickness of 8 μm using a microtome. The tissue sections were blocked with 1% normal goat serum (Jackson ImmunoResearch Laboratories, Inc., West Grove, PA) and then incubated with anti-β-III tubulin (1:500, BioLegend; 801201), anti-Nestin (1:500, Santa Cruz Biotechnology Inc., Dallas, TX, SC-23927), anti-glial fibrillary acidic protein (GFAP) (1:500, Merck Millipore, Burlington, MA, AB5804), anti-Synapsin I (1:500, Merck Millipore; AB1543P), or anti-PSD-95 (1:500, Invitrogen; MA1-046) primary antibody, followed by Alexa Fluor 488- and Alexa Fluor 546-conjugated goat anti-mouse or goat anti-rabbit antibody (1:1000; Thermo Fisher Scientific). Nuclei were labeled with DAPI (1:1000, Sigma-Aldrich). Fluorescence was observed using a Zeiss LSM510 confocal microscope (Carl Zeiss).

### ELISA

For the analysis of soluble Aβ42 levels in WT and AD model mouse brains, brain tissues were homogenized in RIPA buffer (Thermo Fisher Scientific) containing protease inhibitors (GenDEPOT, Barker, TX) and sonicated. The homogenates were then centrifuged at 20,000 × g for 20 min at 4 °C, and supernatant was used for analysis using an Aβ42 ELISA kit (Invitrogen, KHB3441) according to the manufacturer’s instructions. Each experimental sample was tested at least in triplicate.

### Flow cytometry

To analyze the percentage of Muse cells positive for both SSEA3 and CD105 in NTSC populations from the five different donors, single-cell suspensions were prepared, incubated with anti-SSEA3 primary antibody (1:50, Abcam, Cambridge, UK, ab16286) for 1 h at 4 °C, and then with FITC-conjugated goat anti-rat IgM (1:100, Jackson ImmunoResearch) for 30 min at 4 °C. For double staining, the cells were incubated with anti-CD105 (1:100, PE-conjugated, BD Biosciences, San Jose, CA, 560839) for 30 min at 4 °C, resuspended in fluorescence-activated cell sorting (FACS) buffer (Invitrogen), and analyzed by a FACS Canto II instrument (BD Biosciences) with DIVA software.

### Isolation and culture of SSEA3^+^/CD105^+^ NTSCs

To isolate SSEA3^+^/CD105^+^ cells from NTSCs, single-cell suspensions of NTSCs were prepared in FACS buffer (Thermo Fisher Scientific), incubated with anti-SSEA3 primary antibody (1:50, Abcam, ab16286) for 1 h at 4 °C, followed by FITC-conjugated goat anti-rat IgM (1:100, Jackson ImmunoResearch, USA) for 30 min at 4 °C, and subsequently washed with FACS buffer. The cells were then double-stained with anti-CD105 primary antibody (1:100, PE-conjugated, BD Biosciences) for 30 min at 4 °C, washed, and resuspended in FACS buffer. Muse cells that were double positive for SSEA3 and CD105 were isolated by using a FACSCanto II instrument (BD Biosciences) with DIVA software. The collected Muse cells (NTSCs-M) were cultured in α-MEM (Thermo Fisher Scientific) supplemented with 1% (*v*/*v*) antibiotics (Invitrogen) and 10% (*v*/*v*) FBS (Thermo Fisher Scientific) in a 37 °C incubator in 5% (*v*/*v*) CO_2_. To analyze the growth of cultured NTSCs and Muse cells, 9 × 10^3^ cells were plated and incubated for four days, and the absorbance was measured on a microplate reader at a wavelength of 450 nm using an EZ-Cytox assay kit (DAEILLAB Co., Seoul, Korea).

### Cell immunofluorescence staining

The expression of SSEA3 and CD105 in cultured NTSCs was measured by immunofluorescence staining. The cells were fixed with 4% (*w*/*v*) PFA, blocked with 1% (*w*/*v*) normal goat serum, and then incubated with anti-SSEA3 (1:200, Abcam, ab16286) and anti-CD105 (1:200, BD Biosciences, 560839) antibodies and then with an Alexa Fluor 488-conjugated goat anti-rat antibody (Invitrogen). To assess the expression of Nestin and β-III tubulin in NTSCs cultured in neuronal differentiation medium, the cells were fixed with 4% (*w*/*v*) PFA, blocked with 1% (*w*/*v*) normal goat serum, and incubated with anti-Nestin (1:500, Santa Cruz Biotechnology, SC-23927) and anti-β-III tubulin (1:500, BioLegend, 801201) primary antibody followed by Alexa Fluor 488- or Alex Fluor 546-conjugated goat anti-mouse or goat anti-rabbit antibody (1:1000, Thermo Fisher Scientific). Nuclei were labeled with DAPI (1:1000, Sigma-Aldrich), and fluorescence was detected using a Zeiss LSM510 confocal microscope (Carl Zeiss).

### Assessment of multilineage differentiation potential in vitro

The multilineage differentiation potential of human NTSCs was assessed by evaluating the osteogenic, adipogenic, and neurogenic differentiation of the cells. To assess osteogenic differentiation, cells were cultured in osteogenic differentiation medium for 3 weeks, with medium change 3 times a week. On day 21, the cells were fixed with 4% (*w/v*) PFA and stained with 4% (*w/v*) alizarin red S solution to detect calcium deposits as described by Lim et al. [27]. To evaluate adipogenic differentiation, cells were cultured for 3 weeks in adipogenic differentiation medium (Stem Pro®, Thermo Fisher Scientific), with medium change 3 times a week. On day 21, the cells were fixed with 10% (*w/v*) formalin and stained with 0.5% (*w/v*) oil red O solution (Sigma-Aldrich, O0625) to detect lipid droplets. To evaluate neurogenic differentiation, cells were cultured in neurogenic differentiation medium (Thermo Fisher Scientific) for 4 weeks, with medium change 3 times a week. On day 28, the cells were fixed with 4% (*w/v*) PFA and stained for neural markers to detect differentiating or mature neurons. The stained cells were observed under a Zeiss LSM510 confocal microscope.

### Cytokine antibody array

Cytokine secretion profiles were evaluated via the Cytokine Antibody Array Human Cytokine Array C5 Kit (RayBiotech, Peachtree Corners, GA, AAH-CYT-5-2), which contains antibodies against a total of 80 different cytokines. For the analysis of cytokine levels of cultured NTSCs, 5 × 10^5^ NTSCs were cultured in 2–3 mL of medium for 72 h, and the conditioned medium was collected for analysis. To measure cytokine levels of cultured COs, medium conditioned by 4–5 COs (2 × 10^4^ cells per CO) cocultured with or without NTSCs was collected. The array membranes were incubated with conditioned medium and further incubated with a biotin-conjugated antibody cocktail. The membranes were washed, incubated with HRP-conjugated streptavidin, and developed using detection reagent. The arrays were then scanned and analyzed using a computerized imaging system. Optical density was quantified with ImageJ, and the grey values of each spot were averaged across two or three separate membranes.

### Western blotting

Brain tissues or COs were homogenized and sonicated in RIPA buffer (Thermo Fisher Scientific) containing protease inhibitors (GenDEPOT). The homogenates were centrifuged, and supernatants were used for the analysis. Protein samples were loaded onto 4%–12% (*w*/*v*) Bis–Tris protein gels (Thermo Fisher Scientific) and transferred to polyvinylidene difluoride (PVDF) membranes (Roche, Mannheim, Germany). The membranes were incubated with the following primary antibodies: anti-osteopontin (OPN) (1:500, Santa Cruz Biotechnology, SC21742), anti-neprilysin (NEP) (1:1000, Merck Millipore, AB5458), anti-CD11b (1:500, Novus Biologicals, Centennial, CO, NB110-89474), anti-Aβ (6E10, 1:400; BioLegend, 803,002), anti-pTau (1:500, Thermo Fisher Scientific, MN1020), anti-NeuN (1:200, Merck Millipore, ABN78), anti-β-III tubulin (1:500, BioLegend, 801201), anti-Toll-like receptor 4 (TLR4) (1:1000, Proteintech, 19811–1-AP), anti-myeloid differentiation primary response gene-88 (Myd88) (1:1000, Abcam, ab219413), anti-pIkBα (1:1000, Cell Signaling, 2859S), anti-IkBα (1:1000, Abcam, ab32518), anti-NF-kB (phospho S536, 1:1000, ab86299), anti-NF-kB p65 (1:1000, Abcam, ab16502), and anti-β-actin (1:1000, Santa Cruz Biotechnology, SC47778). The membranes were incubated with horseradish peroxidase-conjugated secondary antibodies, and signals were developed using enhanced chemiluminescence detection reagents (Thermo Fisher Scientific).

### Generation and culture of human iPSCs

All experiments involving iPSCs derived from the blood of AD patients were approved by the Institutional Review Board of Yeouido St. Mary’s Hospital (XC22TND10089) and St. Vincent’s Hospital (VC20TIS10005) of the Catholic University of Korea. Informed consent had been obtained from the donors, and the studies were conducted in accordance with the Declaration of Helsinki.

Patient-derived peripheral blood mononuclear cells were isolated via centrifugation (400 × g, 30 min, 4 °C) through a Ficoll gradient and cultured in a 37 °C incubator in 5% (*v/v*) CO_2_ using StemSpan-XF (STEMCELL Technologies, Cambridge, MA; Cat. #100–0073) supplemented with 1% (*v/v*) penicillin/streptomycin (Invitrogen). The cells were reprogrammed with factors Oct4, Sox2, Klf4, and c-Myc using nonintegrative Sendai RNA viruses (CytoTune-iPS Sendai reprogramming Kit, Thermo Fisher Scientific) according to the manufacturer’s instructions. Briefly, 1 × 10^5^ cells were plated in 12-well plates one or two days before transfection and cultured in StemSpan^TM^-XF supplemented with 1% (*v/v*) penicillin/streptomycin (Invitrogen) until the day of transfection. Medium was then replaced with fresh StemSpan-XF containing the CytoTune vector, and the cells were incubated overnight at 37 °C in 5% CO_2_. After overnight incubation, the medium was replaced with fresh StemSpan-XF daily for one week. Seven days after transfection, the cells were harvested and grown on vitronectin-coated plates using mTeSR medium (STEMCELL Technologies; Cat. #85857). Three independent iPSC lines derived from AD patients and two iPSC lines from non-AD patients were used in the experiments. The iPSCs were cultured in Matrigel (Corning, Tewksbury, MA; 354277)-coated dishes in mTeSR1 medium (Stem Cell Technologies, Cambridge, MA; 85850) under feeder-free culture conditions. The iPSCs were passaged every 6–7 days using Accutase (Thermo Fisher Scientific, A1110501) by isolating colonies into clumps and replating them on vitronectin-coated plates. iPSCs derived from the blood of a non-AD control as described by Rim et al. [32] were gifted by Dr Ji Hyeon Ju (The Catholic University of Korea).

### Generation of human COs and treatment

We generated COs from three independent AD patient iPSC lines (AD-COs#1, AD-COs#2, and AD-COs#3) and from two non-AD patient iPSC lines (NAC-COs#1 and NAC-COs#2) using a protocol based on the protocol developed by Lancaster et al. [33]. On days 5–7, 90% confluent iPSCs were dissociated into single cells using Accutase and resuspended in embryoid body formation medium containing 10 μM Y27632 (a Rho-associated protein kinase inhibitor). A total of 1 × 10^4^ cells were plated into each well of low-attachment 96-well, U-bottom plates (Corning) to form a single embryoid body (EB). The EBs were then transferred to neural induction medium (DMEM/F12 supplemented with N2, nonessential amino acids, heparin, and penicillin–streptomycin) and cultured for 5–7 days to promote neural differentiation. Neural progenitor cells were resuspended in Matrigel drops and cultured in differentiation medium (neurobasal medium supplemented with B27, GlutaMAX, and 2-mercaptoethanol). Matrigel drops containing neural progenitor cells were transferred to a spinning bioreactor and cultured in differentiation medium supplemented with EGF (Thermo Fisher Scientific, AF-100-15), FGF2 (Thermo Fisher Scientific, 100-18B), brain-derived neurotrophic factor (BDNF) (Thermo Fisher Scientific, PHC7074), and laminin, and the medium was changed every 3–4 days.

Mature COs, cultured for 40–50 days, were used for the coculture experiments. For each condition, 4–5 COs derived from AD iPSC lines (AD-COs#1–3) or non-AD control iPSC lines (NAC-COs#1–2) were placed in the lower chamber of a Transwell system (Merck Millipore), while NTSCs-H or NTSCs-SC (4–5 × 10^4^ cells) were seeded in the upper chamber. The coculture was maintained for 5–6 days under standard conditions. As a control, mock-treated organoids were maintained in the same culture medium and Transwell setup without the addition of NTSCs. Following coculture, COs and conditioned media were collected for downstream analysis. Donor information for the iPSC-derived COs is as follows: AD-CO#1 and NAC-CO#1 were derived from female donors, whereas AD-CO#2, AD-CO#3, and NAC-CO#2 were derived from male donors. The AD-CO lines were generated from individuals aged 76 (AD-CO#1), 73 (AD-CO#2), and 70 (AD-CO#3) years at the time of iPSC derivation, carrying *APOE3/E4*, *APOE3/E3*, and *APOE4/E4* genotypes, respectively, genetic backgrounds commonly associated with sporadic AD. Both NAC-CO lines were generated from neonatal donors (0 year old at the time of derivation) and carried the *APOE3/E3* genotype.

### Single-cell RNA analysis

To generate each library, four to five organoids were pooled after 6 months of culture, washed twice in PBS, and dissociated with a trypsin-Accutase (1 ×) solution. After dissociation, the reaction was quenched by gradual addition of PBS containing 10% KnockOut^TM^ Serum Replacement (PBS-KnockOut Serum Replacement). The samples were subsequently centrifuged at 400 × g for 5 min at 4 °C, after which the supernatant was removed. The pellet was resuspended in 1–2 mL of PBS-KnockOut^TM^ Serum Replacement and filtered through a 70 μm strainer. The cells were centrifuged again and resuspended in PBS-KnockOut Serum Replacement to a concentration of 450–1000 cells per microliter. Only samples with a cell viability greater than 85% were processed. For each library, 17,000 cells were loaded onto a 10 × Chromium controller, with the aim of recovering 10,000 cells. The libraries were prepared via the Chromium Single Cell 3′ Reagent Kit (version 3.1) and sequenced on NovaSeq S4 flow cells with the aim of obtaining more than 20,000 reads per cell. The raw sequencing reads were aligned to the GRCh38 reference genome using CellRanger v.7.2.0. Doublets were identified and removed from each library using Scrublet v.0.2.3 [34]. Downstream analysis was conducted using the Seurat [35] R package v.5.0.1 (in R v.4.2.1). Quality control involved excluding cells with fewer than 500 or more than 10,000 detected genes, a total count below 1500, or more than 10% mitochondrial gene reads. The raw counts were normalized via SCTransform [36]. The mitochondrial gene percentage and cell cycle score were regressed out via the CellCycleScoring function. The top 2000 highly variable genes were selected for principal component analysis. Integration of the single-cell RNA sequencing (scRNA-seq) data was performed via the Harmony [37] R package v.1.2.0 (in R v.4.2.1). The cells were clustered via the FindClusters function on the basis of the Louvain algorithm with a resolution of 1.0. UMAP embedding was achieved via the RunUMAP function with 40 dimensions. The marker genes for each cluster were identified via the FindAllMarkers function. Differential gene expression analysis was performed using DESeq2 (v1.48.1) in R (v4.5.1) under a pseudobulk design, aggregating gene counts from all cells within each condition (AD vs. non-AD). For gene set enrichment analysis (GSEA), genes were ranked by log_2_ fold change, and enrichment was assessed using the msigdbr (v24.1.0) and fgsea (v1.34.0) packages in R (v4.5.1). Gene sets containing 25–500 genes were included in the analysis. The Single-cell RNA-seq data of this study are available on the BioProject with accession ID PRJNA1151219 (The datasets generated during the current study are available via a reviewer-only access link during the peer-review process. https://dataview.ncbi.nlm.nih.gov/object/PRJNA1151219?reviewer=mprp6mfhhqdi9k682cuetb6o19)**.**

### Calcium imaging in COs

Calcium imaging experiments were conducted using COs at 40 days of differentiation. Fluo-4 AM dye (Thermo Fisher Scientific; F14201) was freshly prepared by dissolving a 50-µg vial in 44 µL of DMSO to obtain a 1 mM stock solution. To enhance dye uptake, Fluo-4 AM was mixed with 20% (*w/v*) Pluronic F-127 in DMSO (Thermo Fisher Scientific; P3000MP). A working solution containing 2 µM Fluo-4 AM and Pluronic F-127 was prepared by adding 4 µL of the Fluo-4 AM stock to 1 mL of prewarmed CO medium. Organoids were incubated in the loading solution at 37 °C with 5% CO_2_ for 30 min in the dark, followed by a single wash with prewarmed medium. Subsequently, organoids were incubated in dye-free medium for 20 min to allow for de-esterification. Calcium imaging was performed using a Zeiss LSM900 confocal laser scanning microscope equipped with a FITC filter set. Organoids were placed in confocal imaging dishes and maintained at 37 °C with 5% CO_2_ during image acquisition. Background fluorescence was subtracted from each region of interest at every time point.

### Quantification and statistical analysis

All data are expressed as the mean (SD) from at least three independent experiments. Tukey’s post hoc ANOVA was performed to compare data from multiple groups. *P* < 0.05 was considered to be statistically significant. For each animal, multiple brain sections (3 sections per mouse) were analyzed, and values were averaged to obtain one representative data point per animal. No data or samples were excluded unless sections were damaged or of insufficient quality for staining and imaging. To analyze the amyloid plaque areas, immunofluorescence-positive regions in six randomly selected, nonoverlapping, and anatomically matched regions in each brain section (three to five animals per group) were analyzed using the ZEN imaging software (Carl Zeiss). To quantify Iba-1-, NeuN-, TUNEL-, and HuNu-positive cells in brain tissue, cells in 5–6 randomly selected, nonoverlapping and similar regions per section (three to five animals per group) were counted. Stained cells were counted using the Image-Pro Plus software (Media Cybernetics, Inc., Rockville, MD; http://www.mediacy.com).

## Results

### NTSCs from different donors improve cognitive impairment and AD-associated neuropathological changes to varying degrees in the 5 × FAD AD mouse model

**Fig. 1:**
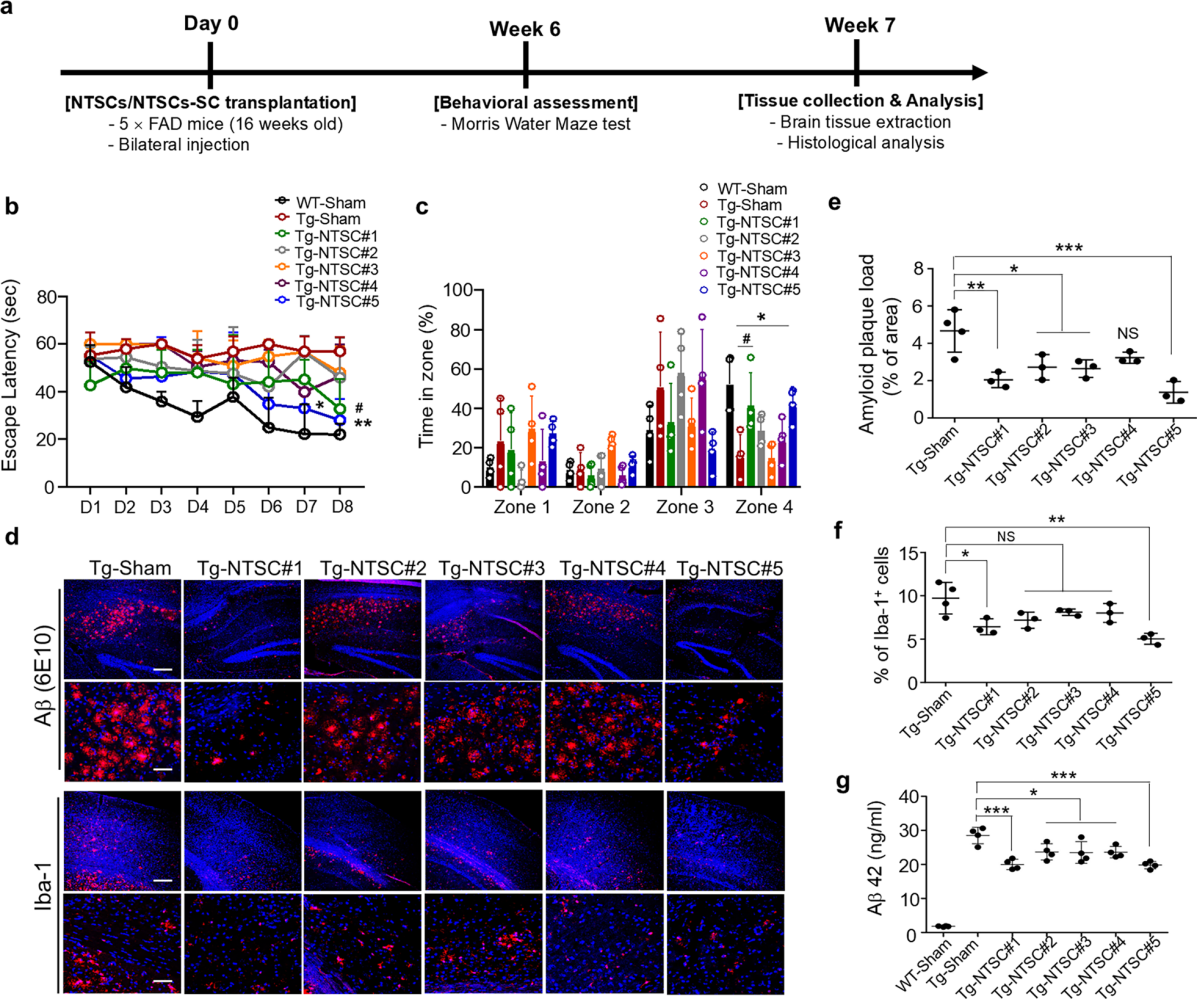
Effects of NTSCs from different donors on cognitive function and neuropathology in Tg AD mice. **a** Schematic diagram of in vivo experimental timeline. **b** Escape latency in the Morris water maze. Two-way ANOVA was used to determine the significance of differences among multiple groups (*n* = 4 per group), followed by Tukey’s post hoc test for multiple comparisons. *P < 0.05 and **P < 0.01 vs the Tg − NTSC#5 group on days 7 and 8, ^#^*P* < 0.05 vs the Tg-NTSC#1 group on day 8. **c** The percentage (%) of time spent in each zone over 60 s in the probe test (platform location was in zone 4). Two-way ANOVA was used to determine the significance of differences among multiple groups (n = 4 per group), followed by Tukey’s post hoc test for multiple comparisons*P < 0.05 vs the Tg − NTSC#5 group, ^#^*P* < 0.05 vs the Tg-NTSC#1 group. **d** Confocal microscopy images of OCT-embedded brain sections from the Tg-sham, Tg-NTSC#1, Tg-NTSC#2, Tg-NTSC#3, Tg-NTSC#4, and Tg-NTSC#5 groups after staining with the 6E10 antibody to detect Aβ deposition and an anti-Iba-1 antibody to detect microglia at 7 weeks post-transplantation. Nuclei were labeled with DAPI (blue). Scale bars: 100 μm (main image) and 50 μm (magnified views). **e** The 6E10-positive areas in the hippocampus and cortex were quantified (*n* = 3 − 4 per group). The values are the means (SDs). **f** Quantification of the number of Iba-1-positive cells in the hippocampus and cortex (*n* = 3 − 4 per group). The values are the means (SDs). **g** Aβ42 levels in brain tissue lysates of mice analyzed via ELISA (*n* = 4 per group). The images and data are representative of two or three independent experiments. **e**–**g**, One-way ANOVA was used to determine the significance of differences among multiple groups, followed by Tukey’s post hoc test. * P < 0.05, ** P < 0.01, and *** P < 0.001

MSCs derived from different donors have demonstrated significant variability in the in vitro characteristics and in vivo therapeutic efficacy [22–24]. To investigate whether the effects of NTSC transplantation on cognitive function in AD vary depending on the donor, we injected PBS (sham) or NTSCs from one of five healthy donors (NTSCs#1–5) into the hippocampus of 16-week-old 5 × FAD Tg mice (Fig. 1a). Six weeks post-transplantation, learning and memory abilities were assessed by the MWM test. In the 8-day training session, the escape latency to find the target platform progressively decreased in the WT-sham and Tg-NTSC groups, while no significant improvement was observed in the Tg-sham group. Among the Tg mice injected with NTSCs from the 5 donors, the Tg-NTSC#1 group mice showed significantly shorter escape latency than the Tg-sham group mice on day 8 (*P* < 0.05). The Tg-NTSC#5 group mice showed significantly shorter escape latency than the Tg-sham group mice from day 7 (*P* < 0.05), one day earlier than the Tg-NTSC#1 group mice. However, the Tg-NTSC#2, Tg-NTSC#3, and Tg-NTSC#4 group mice did not exhibit a significant decrease in escape latency during the 8 days, compared with the Tg-sham group mice (Fig. 1b). Detailed quantitative data of average escape latency in each group are provided in Additional file 2.

In the probe test, the WT-sham, Tg-NTSC#1, and Tg-NTSC#5 group mice spent significantly more time in the target quadrant (zone 4) than did the Tg-sham group mice. However, the Tg-NTSC#2, Tg-NTSC#3, and Tg-NTSC#4 groups did not show significant difference from the Tg-sham group (Fig. 1c). These results demonstrated that NTSC transplantation improved learning and memory abilities, but this effect differed among the NTSC donors.

Next, we explored whether the observed differences in learning and memory were associated with variations in the ability of NTSCs from different donors to reduce plaque deposition and inflammation. Fluorescence immunohistochemistry at 7 weeks post-transplantation revealed a substantial Aβ plaque burden and an elevated number of inflammatory microglia in the hippocampal and cortical regions of Tg-sham group mice. These pathological markers were significantly reduced in the Tg-NTSC group mice (Fig. 1d). Among the Tg-NTSC groups, the Tg-NTSC#5 group exhibited the most significant decrease in both Aβ plaque burden and the number of Iba-1-positive microglia. Detailed quantitative data for Aβ plaque load and Iba-1-positive microglia are provided in Additional file 2. In addition, the soluble Aβ42 levels in brain homogenates were significantly lower in the Tg-NTSC group compared to the Tg-sham group, with the greatest reductions observed in the Tg-NTSC#1 and Tg-NTSC#5 groups (Fig. 1g). These findings suggest that NTSCs from different donors improve cognitive impairment and AD-associated neuropathological changes to varying degrees in the 5 × FAD AD mouse model (Table S5).

### Characteristics of NTSCs with a higher percentage of muse cells (NTSCs-H) and muse cells isolated from NTSCs (NTSCs-SC)

**Fig. 2:**
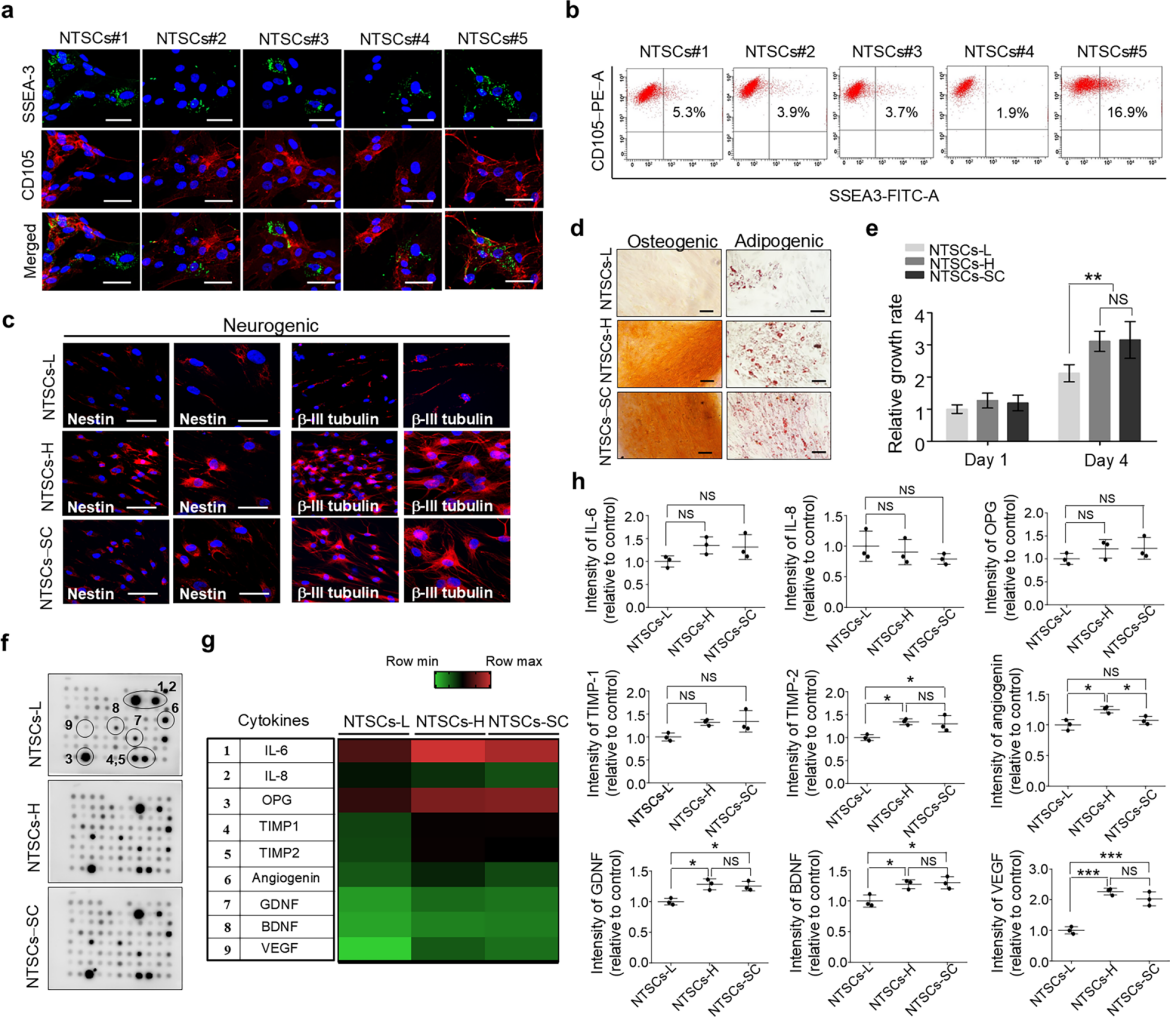
Characteristics of NTSCs-L, NTSCs-H, and NTSCs-SC in culture. **a** Confocal microscopy images of NTSCs#1, NTSCs#2, NTSCs#3, NTSCs#4, and NTSCs#5 cultured in proliferation medium after double staining for SSEA3 (green) and CD105 (red). Scale bars: 20 μm. **b** Flow cytometry analyses of SSEA3 and CD105 expression. **c** Confocal microscopy images of nestin and β-III tubulin staining in NTSCs with the lowest percentage of Muse cells at passage 5 (NTSCs-L), those with the highest percentage at passage 5 (NTSCs-H), and Muse cells isolated from NTSCs and subsequently expanded to passage 5 (NTSCs-SC), after 28 days of incubation in neurogenic differentiation medium. Scale bars: 100 μm (left panel) and50 μm (right panel). **d** Images of cultured NTSCs-L, NTSCs-H, and NTSCs-SC stained with alizarin red S and oil red O. Scale bars: 50 μm. **e** Relative growth rates of NTSCs-L, NTSCs-H, and NTSCs-SC incubated in proliferation medium at 4 days after plating. Statistical analysis was performed using two-way ANOVA followed by Tukey’s post hoc test. **P < 0.01. **f** Cytokine secretion profiles of NTSCs-L, NTSCs-H, and NTSCs-SC in culture. **g** Heatmap was generated to visualize the cytokine secretion profiles of NTSCs-L, NTSCs-H, and NTSCs-SC based on array membrane analysis. **h** Gray values of IL-6, IL-8, OPG, TIMP-1/2, angiogenin, GDNF, BDNF, and VEGF spots on the array membrane. The values shown are the means (SDs). Statistical analysis was performed using one-way ANOVA followed by Tukey’s post hoc test. *P < 0.05, ***P < 0.001. The images and data are representative of two or three independent experiments

To investigate whether the efficacy of NTSCs correlates with the presence of Muse cells (SSEA3^+^/CD105^+^), we quantified their percentage within NTSCs from different donors. Immunostaining and flow cytometry showed varying percentages of Muse cells among the NTSCs from different donors (Fig. 2a, b). The percentages of Muse cells (SSEA3^+^/CD105^+^) in the NTSCs#1, NTSCs#2, NTSCs#3, NTSCs#4, and NTSCs#5 were approximately 5.3%, 3.1%, 3.0%, 1.8%, and 16.9%, respectively. Notably, the NTSC#5, which contained the highest percentage of Muse cells (SSEA3^+^/CD105^+^), showed the greatest efficacy in improving AD-related pathology and cognitive deficits.

We next compared the in vitro properties of NTSCs with the highest percentage of Muse cells at passage 5 (NTSCs-H), those with the lowest percentage at passage 5 (NTSCs-L), and Muse cells isolated from NTSCs and subsequently expanded up to passage 5 (NTSCs-SC). When cultured under neuronal differentiation conditions, both NTSCs-H and NTSCs-SC showed robust expression of neuronal progenitor markers Nestin and β-III tubulin at 4 weeks (Fig. 2c), while NTSCs-L showed weak expression. Furthermore, under osteogenic and adipogenic differentiation conditions, both NTSCs-H and NTSCs-SC showed stronger staining with alizarin red S and oil red O at 2–3 weeks when compared with NTSCs-L (Fig. 2d). During the 4-day culture period, both NTSCs-H and NTSC-SC exhibited an approximately 1.5-fold faster growth rate than NTSCs-L, while there was no significant difference between NTSCs-H and NTSCs-SC (Fig. 2e).

Cytokine secretion analysis using the human cytokine array kit containing antibodies against 80 different cytokines showed strong signals for MSC-related cytokines, such as interleukin (IL)-6, IL-8, tissue inhibitor of metalloproteinase-1 (TIMP-1), TIMP2, osteoprotegerin (OPG), angiogenin, vascular endothelial growth factor (VEGF), BDNF, and glial cell derived neurotrophic factor (GDNF) among the three groups (Fig. 2f, g). To analyze the data, signal intensities of corresponding spots were quantified and averaged across three independent array membranes. The levels of TIMP-2, VEGF, BDNF, and GDNF were significantly higher in both NTSCs-H- and NTSCs-SC-conditioned media compared to NTSCs-L-conditioned media. These cytokines are known to promote stem cell functions such as proliferation and differentiation, provide neuroprotection, support neurogenesis, and enhance immunomodulation, thereby contributing to improved therapeutic outcomes in neurological disease models, including AD [38–42]. However, no significant difference was observed between NTSCs-H and NTSCs-SC (Fig. 2h). These findings indicate that NTSCs-H have comparable stem cell properties in vitro compared to NTSCs-SC.

### Transplantation of NTSCs-H or NTSCs-SC reduces Aβ plaque deposition and improves cognitive function in 5 × FAD Tg mice

**Fig. 3:**
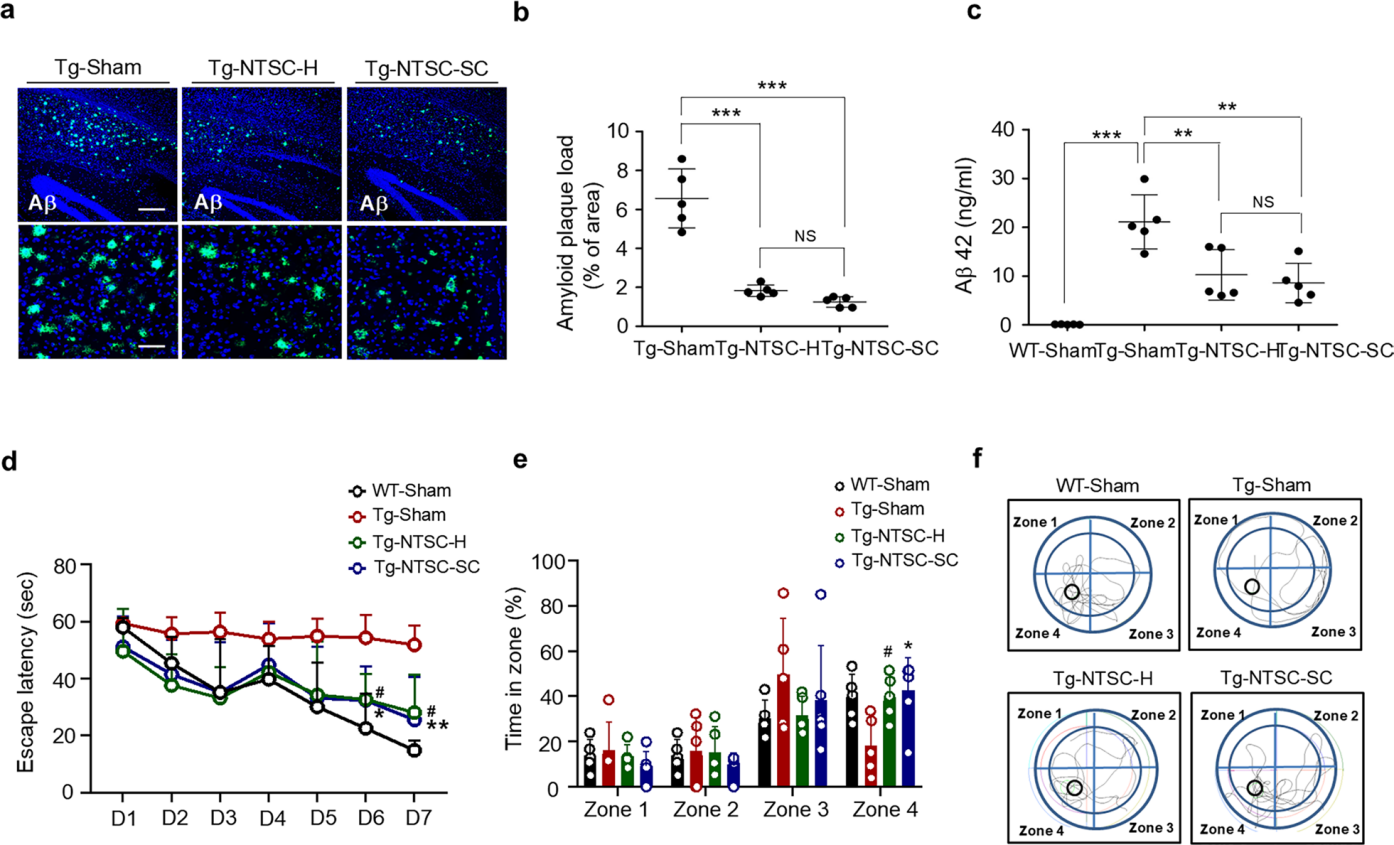
Effects of NTSCs-H or NTSCs-SC on Aβ load and cognitive impairment in Tg AD mice. **a** Confocal microscopy images of OCT-embedded brain sections from mice in the Tg-sham, Tg-NTSC-H, and Tg-NTSC-SC groups after staining with the 6E10 antibody to detect Aβ deposition. Scale bars: 200 μm (main image) and 50 μm (magnified views). **b** The 6E10-positive areas in the hippocampus and cortex were quantified (*n* = 5 per group). The values are the means (SDs). **c** Aβ42 levels in brain tissue lysates from mice were analyzed via ELISA (*n* = 5 per group). For both **b** and** c**, statistical analysis was performed using one-way ANOVA followed by Tukey’s post hoc test, **P < 0.01 and ***P < 0.001. **d** Escape latency in the Morris water maze was analyzed using two-way ANOVA with Tukey’s post hoc test (*n* = 5–6 per group). **P < 0.01 vs the Tg-NTSC-SC group and ^#^*P* < 0.05 vs the Tg-NTSC-H group on day 7. **e** The percentage (%) of time spent in each zone over 60 s. Statistical analysis was performed using two-way ANOVA followed by Tukey’s post hoc test (*n* = 5–6 per group). *P < 0.05 vs the Tg-NTSC-SC group and ^#^*P* < 0.05 vs the Tg-NTSC-H group. **f** Images of trajectories in the probe test. All images and data are representative of two or three independent experiments

PBS, NTSCs-H, or NTSCs-SC were injected into the brains of 16-week-old 5 × FAD Tg mice. At 7 weeks post-transplantation, Aβ plaque deposition was significantly reduced in mice receiving NTSCs-H or NTSCs-SC (Fig. 3a, b). Detailed quantitative data for Aβ plaque load are provided in Additional file 2. Additionally, ELISA analysis indicated that the soluble Aβ42 levels in brain homogenates were significantly reduced by approximately 2.0–2.5 folds in the Tg-NTSC-H and Tg-NTSC-SC groups compared to the Tg-sham group (Fig. 3c). However, no significant differences in Aβ42 levels were observed between the Tg-NTSC-H and Tg-NTSC-SC groups. These findings suggest that NTSCs-H and NTSCs-SC are equally effective in reducing Aβ plaque deposition and soluble Aβ42 levels.

Additionally, in the MWM test at 6 weeks post-transplantation, the WT-sham, Tg-NTSC-H, and Tg-NTSC-SC group mice had a shorter escape latency than the Tg-sham group mice on day 7 of training (Fig. 3d). Compared with the Tg-NTSC-H group, the Tg-NTS-SC group showed an even greater decrease in escape latency on day 7 of training. Detailed quantitative data for escape latency are provided in Additional file 2.

In the probe test, mice in the WT-sham, Tg-NTSC-H, and Tg-NTSC-SC groups spent significantly more time in the target quadrant (zone 4) than did mice in the Tg-sham group. There was no significant difference in time spent in the target quadrant between the Tg-NTSC-SC and Tg-NTSC-H groups (Fig. 3e, f). Detailed quantitative data for time spent in the target quadrant are provided in Additional file 2.

### Transplantation of NTSCs-H and NTSCs-SC decreases neuroinflammation and protects against neuronal death in 5 × FAD Tg mice

**Fig. 4:**
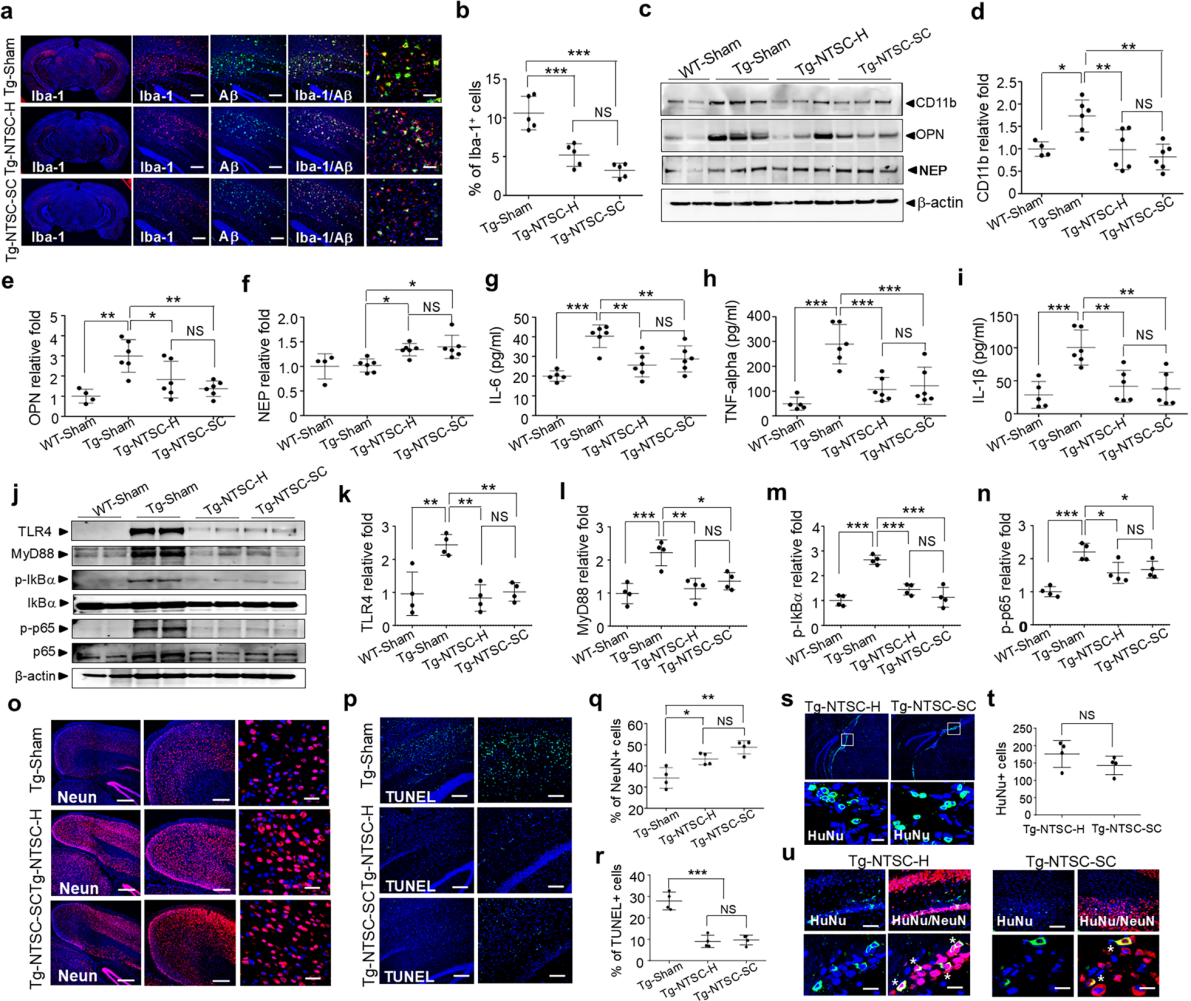
Effects of NTSCs-H or NTSCs-SC on neuro-inflammation and neuronal death in Tg AD mice. **a** Confocal microscopy images of OCT-embedded brain sections from mice in the Tg-sham, Tg-NTSC-H, and Tg-NTSC-SC groups after double staining with the 6E10 antibody and an antibody against Iba-1 at 7 weeks post-transplantation. Scale bars 200 μm (left) and 50 μm (rightmost). **b** Quantification of the number of Iba-1-positive cells in the hippocampus and cortex in each group (*n* = 5 per group). The values are the means (SDs). **c-f** Western blot analysis of CD11, OPN, and NEP in brain tissue lysates at 7 weeks post-transplantation, and quantification of protein levels. β-Actin was used as a loading control. **g-i** ELISA analysis of levels of the proinflammatory cytokines IL-6, TNF-α, and IL-1β in the brain tissue lysates at 7 weeks post-transplantation (*n* = 6 per group). **j-n** Western blot analysis of TLR4, MyD88, p-IkBα, IkBα, p-p65 NF-kB, and p65 NF-kB in brain tissue lysates at 7 weeks post-transplantation, and quantification of protein levels. **o, p** Confocal microscopy images of OCT-embedded brain sections from Tg-sham, Tg-NTSC-H, and Tg-NTSC-SC group mice after staining with an anti-NeuN antibody and TUNEL assay kit at 7 weeks post-transplantation. Scale bars: **o**, 500 μm (left); 200 μm (middle); 20 μm (right); **p**, 200 μm (left); 100 μm (right). **q, r** Quantification of NeuN-positive cells and TUNEL-positive cells in the hippocampus and cortex (*n* = 4 per group). **s** Confocal microscopy images of OCT-embedded brain sections from Tg-NTSC-H and Tg-NTSC-SC group mice after staining with an anti-HuNu antibody. Scale bar: 10 μm. **t** HuNu-positive cells were counted in the brains of Tg-NTSC-H and Tg-NTSC-SC group mice (*n* = 4 per group). Values are the mean (SD). **u** Confocal microscopy images of brain sections with double staining for HuNu (green) and NeuN (red) at 7 weeks post-transplantation. Nuclei were labeled with DAPI (blue). Scale bars: 50 μm (upper) and 10 μm (lower). All images and data are representative of two or three independent experiments. Statistical analysis was performed using one-way ANOVA followed by Tukey’s post hoc test (**b**, **d**, **e**-**i**, **k**-**n**, **q**, **r**), or Student’s *t*-test (**t**). *P < 0.05, **P < 0.01, and ***P < 0.001

PBS, NTSCs-H at passage 5 and NTSCs-SC, which represent Muse cells purified from NTSCs and subsequently expanded to passage 5, were stereotactically injected into the brains of 16-week-old 5 × FAD Tg mice. Aβ plaque deposition and neuroinflammation were analyzed at 7 weeks post-transplantation by immunostaining with antibody 6E10 and anti-Iba-1. Confocal microscopy showed that a large number of microglia were concentrated around or embedded in Aβ plaques in the brains of Tg-sham group mice, whereas NTSCs-H and Tg-NTSCs-SC both decreased the numbers of Aβ plaques and Iba-1-positive microglia (Fig. 4a). The percentage of Iba-1-positive cells in the brain was significantly reduced by ~ 2.0–3.0 folds in the Tg-NTSC-H and Tg-NTSC-SC groups compared to the Tg-sham group (Fig. 4b). However, no significant difference was observed between the Tg-NTSC-H and Tg-NTSC-SC groups.

Western blotting revealed that the levels of the microglial marker CD11b were approximately 1.8-fold higher in the Tg-sham group compared to the WT-sham group. The CD11b levels were significantly reduced by approximately 1.8 and 2.1 folds in the Tg-NTSC-H and Tg-NTSC-SC groups, respectively, with no significant difference between the two groups (Fig. 4c, d). OPN, a multifunctional inflammatory cytokine implicated in cell-mediated immunity, inflammation, and neurodegenerative diseases [43], was significantly higher in the Tg-sham group than in the WT-sham group, and significantly reduced in the Tg-NTSC-H and Tg-NTSC-SC groups, compared to the Tg-sham group. There was no significant difference between the Tg-NTSC-H and Tg-NTSC-SC groups (Fig. 4c, e).

Previous studies suggest that the impaired ability of neurons to clear Aβ generated by dysfunctional cleavage enzymes can lead to the progression of AD neuropathology through Aβ accumulation and neuroinflammation [44, 45]. To investigate this, we analyzed protein levels of NEP, a key regulator of Aβ degradation and clearance, by Western blotting. The NEP levels increased significantly in the Tg-NTSC-H and Tg-NTSC-SC groups compared to the Tg-sham group, with no significant difference between the former two groups (Fig. 4c, f).

To further examine whether transplantation of NTSCs-H or NTSCs-SC modulates neuroinflammation, inflammatory cytokine levels in brain extracts were measured at 7 weeks post-transplantation. ELISA results revealed that the levels of proinflammatory cytokines IL-6, TNF-α, and IL-1β were significantly elevated by approximately 2–3 folds in the Tg-sham group compared to the WT-sham group. These levels were significantly reduced in both the Tg-NTSC-H and Tg-NTSC-SC groups, with no significant difference between the two groups (Fig. 4g–i). Collectively, these findings suggest that NTSC transplantation reduces neuroinflammation and promotes Aβ clearance, without eliciting a significant immune response.

Nuclear factor-kB (NF-κB) is a key transcription factor regulating innate and adaptive immune responses by inducing expression of pro-inflammatory genes and activating inflammasomes. In AD, NF-κB signaling contributes to microglial activation, leading to Aβ42 accumulation and increased production of pro-inflammatory cytokines, thereby promoting disease pathogenesis [46–48]. We investigated the expression levels of TLR4 and Myd88, which are key upstream regulators of NF-κB signaling that drive the secretion of proinflammatory cytokines such as IL-1β, IL-6, and TNF-α, and are also implicated in the regulation of microglial phenotypic polarization [49]. Western blot analysis revealed that the protein levels of TLR4 and Myd88 were approximately 2.3-fold higher in the Tg-sham group compared to the WT-sham group. Their levels were reduced significantly by approximately 2 folds in both the Tg-NTSC-H and Tg-NTSC-SC groups compared to the Tg-sham group, with no significant difference observed between the two groups (Fig. 4j–l). Similarly, the phosphorylation levels of IkB and p65 NF-kB were markedly increased in the Tg-sham group compared to the WT-sham group. These elevated phosphorylation levels were significantly reduced in both Tg-NTSC-H and Tg-NTSC-SC groups, with no significant difference observed between the two groups (Fig. 4j, m, n).

We further investigated the protective effects of NTSC-H and NTSC-SC transplantation against neuronal death in AD. Immunofluorescence staining of brain sections revealed that the average percentage of NeuN-positive cells in the cortical region at 7 weeks post-transplantation increased significantly by approximately 1.3–1.4 folds in the Tg-NTSC-H and Tg-NTSC-SC groups compared to the Tg-sham group (Fig. 4o, q). Furthermore, TUNEL staining showed a marked presence of TUNEL-positive cells in the cortical and hippocampal regions of Tg-sham mice. However, the number of TUNEL-positive cells was significantly reduced in both the Tg-NTSC-H and Tg-NTSC-SC groups at 7 weeks after transplantation (Fig. 4p, r). Detailed quantitative data for TUNEL-positive cells are provided in Additional file 2. These findings suggest that NTSC-H and NTSC-SC transplantation significantly inhibited neuronal loss in the brains of AD model mice. However, there was no significant difference in the percentage of NeuN- and TUNEL-positive cells between the Tg-NTSC-H and Tg-NTSC-SC groups. Immunofluorescence staining for HuNu confirmed the presence of transplanted human cells around the injection site, as well as in the cortical and hippocampal regions of Tg-NTSC-H and Tg-NTSC-SC mice at 7 weeks post-transplantation (Fig. 4s, t). In addition, immunofluorescence staining revealed that a subset of cells were positive for both HuNu and NeuN (Fig. 4u), suggesting that the engrafted human cells had differentiated into neurons in both the Tg-NTSC-H and Tg-NTSC-SC groups. Collectively, these results indicate that transplantation of NTSCs-H and NTSCs-SC effectively suppresses inflammation and protects neuronal cells in the brains of AD model mice.

### Characteristics of COs derived from iPSCs of three different AD patients

**Fig. 5:**
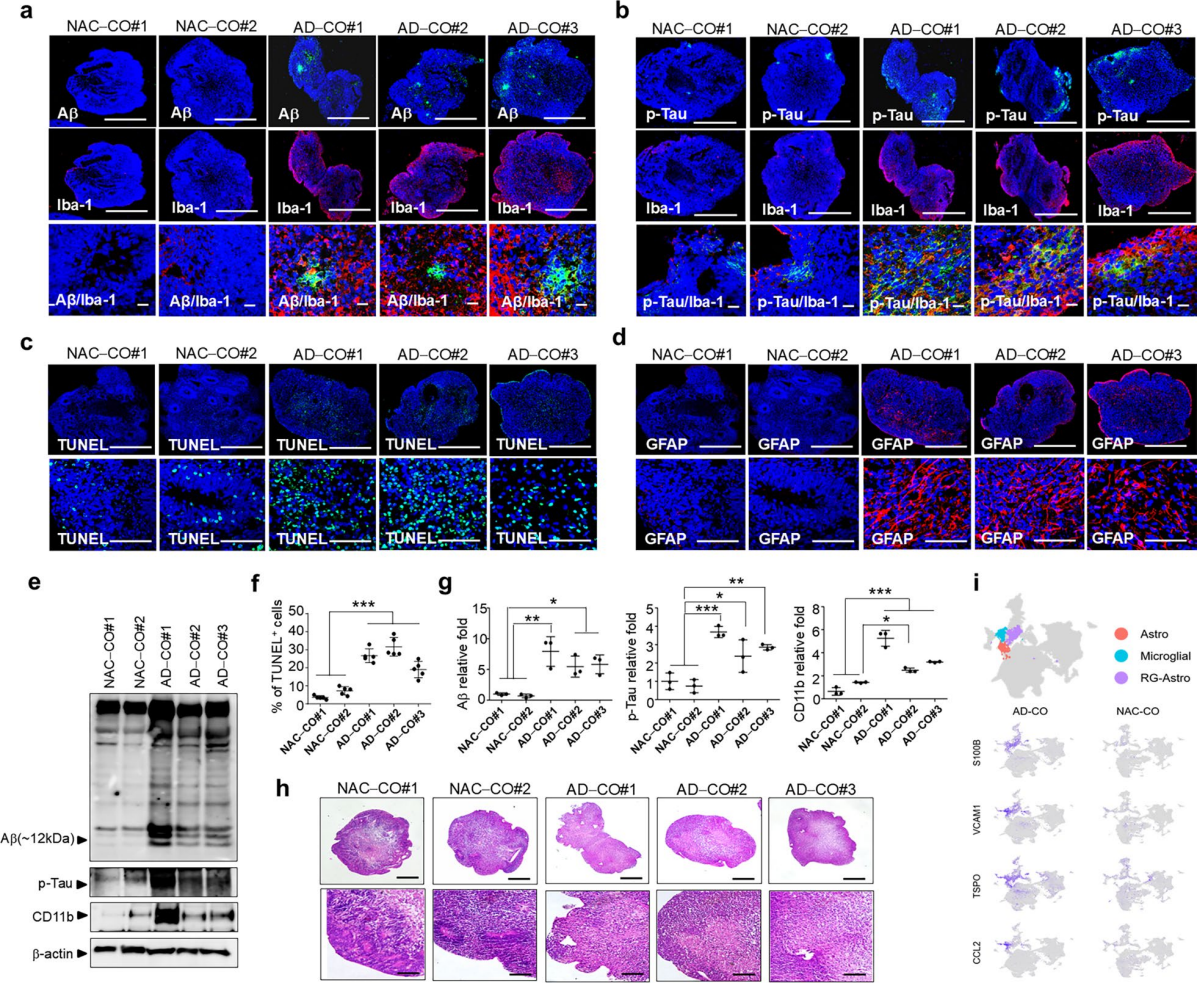
Characteristics of COs derived from iPSCs of three different AD patients (AD-COs#1–3). **a, b** Confocal microscopy images of paraffin-embedded sections with staining for the Aβ aggregate-specific antibody 6E10, the microglial marker Iba-1, and p-tau (Ser202/Thr205). Nuclei were stained with DAPI (blue). Scale bars: 500 μm (upper and middle) and 20 μm (lower). **c, d** Confocal microscopy images of paraffin-embedded sections with TUNEL staining and reactive astrocyte marker GFAP staining. Nuclei were labeled with DAPI (blue). Scale bars: 200 μm (upper) and 20 μm (lower). **e** Western blot analysis of proteins extracted from AD-COs#1 − 3 and NAC-COs#1–2. **f** Quantification of TUNEL-positive cells in NAC-COs#1–2 and AD-COs#1–3 (*n* = 5 per group). The values are the means (SDs). **g** Quantification of the Western blot results. **h** H&E staining of NAC-COs#1–2 and AD-COs#1–3. Scale bars: 100 μm (upper) and 200 μm (lower). **i** Uniform manifold approximation and projection (UMAP) plot showing integrated scRNA-seq data from 78,106 cells derived from AD-COs and NAC-COs. A total of 33 cell types were identified. Each point in the plot represents a single cell. The lower UMAP plots display the expression levels of *S100B*, *VCAM1*, *TSPO,* and *CCL2* in AD-COs and NAC-COs, with darker colors indicating higher expression levels. Statistical analysis was performed using one-way ANOVA followed by Tukey’s post hoc test, *P < 0.05, **P < 0.01, and ***P < 0.001

The efficacy of NTSCs-H and NTSCs-SC in mitigating neuropathological changes was further evaluated using a CO model that closely recapitulates AD. COs were generated from iPSCs derived from the blood of three sporadic AD patients (designated as AD-COs#1, AD-COs#2, and AD-COs#3) and from two non-AD control iPSC lines (designated NAC-COs#1 and NAC-COs#2). Immunofluorescence staining with the 6E10 antibody and an anti-Iba-1 antibody revealed that microglial cells were clustered around the Aβ aggregates in AD-COs#1–3. The Aβ-positive aggregates displayed heterogeneity in size, indicating variable levels of Aβ aggregation among the three AD-COs. In contrast, Aβ aggregates were absent in NAC-COs#1–2. There were fewer Iba-1-positive cells in NAC-COs compared to AD-COs (Fig. 5a). In addition, there was a higher abundance of p-tau (Ser202/Thr205)-positive cells in AD-COs#1–3 compared to NAC-COs#1–2. Notably, p-tau expression levels varied among the COs derived from the three AD patients (Fig. 5b). TUNEL staining showed a marked increase in apoptotic cell death in AD-COs#1–3, with the number of TUNEL-positive cells approximately 2.5–5.0-fold higher than that observed in NAC-COs#1–2 (Fig. 5c, f). Astrogliosis, a pathological hallmark of AD, is characterized by proliferation and activation of astrocytes, typically indicated by elevated expression of GFAP [50, 51]. In reaction to AD pathology, astrogliosis is upregulated [52, 53], contributing to disease progression by impairing the clearance of Aβ and hyperphosphorylated tau, thereby exacerbating neuroinflammation [54]. Immunofluorescence staining using an anti-GFAP antibody revealed an increased number of reactive astrocytes in AD-COs#1–3. In contrast, the GFAP-positive reactive astrocytes were absent in NAC-COs#1–2 (Fig. 5d). Collectively, these findings demonstrate that AD-COs#1–3, but not NAC-COs#1–2, recapitulate key AD-related pathological features, including Aβ aggregation, tau phosphorylation, apoptosis, inflammation, and astrogliosis. Moreover, the extent of these pathological changes varied among the COs derived from different AD patient iPSCs, highlighting inter-individual variability in disease pathology.

To quantitatively assess levels of AD-related proteins, Western blot analysis was performed. Immunoblotting with the 6E10 antibody revealed multiple Aβ-specific bands in AD-COs#1–3, which were absent in NAC-COs#1–2 (Fig. 5e). Analysis of the 12 kDa isoform of Aβ, an isoform strongly associated with cognitive impairment and neurotoxicity [55], showed that Aβ protein levels varied among AD-COs#1–3 but were significantly elevated by approximately 5.0 to 7.0 folds compared to NAC-COs#1–2 (Fig. 5e, g). Similarly, the levels of p-tau and CD11b were markedly increased by 2.5–4.0 folds and 2.5–5.0 folds, respectively, in AD-COs#1–3 (Fig. 5e, g). Hematoxylin and eosin (H&E) staining showed multiple neural rosette-like structures in NAC-COs#1–2, indicative of neuroprogenitor development, whereas these structures were absent in AD-COs#1–3 (Fig. 5h). These findings suggest that NAC-COs#1–2 displayed complex morphology with heterogeneous neural rosette structures, which were absent in AD-COs#1–3.

To further investigate cellular differences between COs derived from normal controls and AD patients, scRNA-seq was performed. A total of 78,106 cells were analyzed and clustered into 41 distinct groups (Fig. S1a, b). Cell type annotation identified 28 distinct cell types after excluding the “Undefined” category (Fig. S2a). Annotations were made based on canonical marker expression in combination with cluster-specific marker gene profiles.

Consistent with previous studies of AD COs [52], progenitor cell types in NAC-COs exhibited higher expression levels of Notch signaling genes, including *HES1*, *HES4*, *HES5*, and *FABP7*, with *Wnt-RG* being the only exception (Fig. S2b). In contrast, AD-COs showed an increased proportion of immature and mature neurons and a decreased proportion of retinal progenitor cells (Fig. S2c).

To further characterize molecular differences between AD-COs and NAC-COs, differential gene expression (DEG) analysis and GSEA were performed. Results revealed significant enrichment of AD-associated pathways in AD-COs (Fig. S2d; Tables S1, S2), including “antigen processing and presentation of exogenous peptide antigen via MHC class II”, “antigen processing and presentation of peptide or polysaccharide antigen via MHC class II”, and “TNF-α signaling via NF-κB” [56–58].

Furthermore, cell type-specific DEG analyses of astrocyte and microglial clusters demonstrated significant upregulation of neuroinflammation-related genes in AD-COs compared with NAC-COs, including *VCAM1*, *TSPO*, and *CCL2*, with *S100B* showing specific upregulation in microglia (Fig. 5i and Table S3, S4). These genes are well-established mediators of neuroinflammation and have been implicated in the pathogenesis of AD [59–63].

Collectively, the results from immunostaining, Western blotting, and scRNA-seq analyses demonstrate that AD-COs, but not NAC-COs, recapitulate key neuropathological features observed in AD patients. The elevated expression of neuroinflammatory genes in AD-COs further highlights their potential as a valuable model system for investigating the therapeutic efficacy of NTSCs in the treatment of AD.

### Validation of NTSCs-H and NTSCs-SC modulation of AD-related pathological changes in AD-COs#1–3

**Fig. 6:**
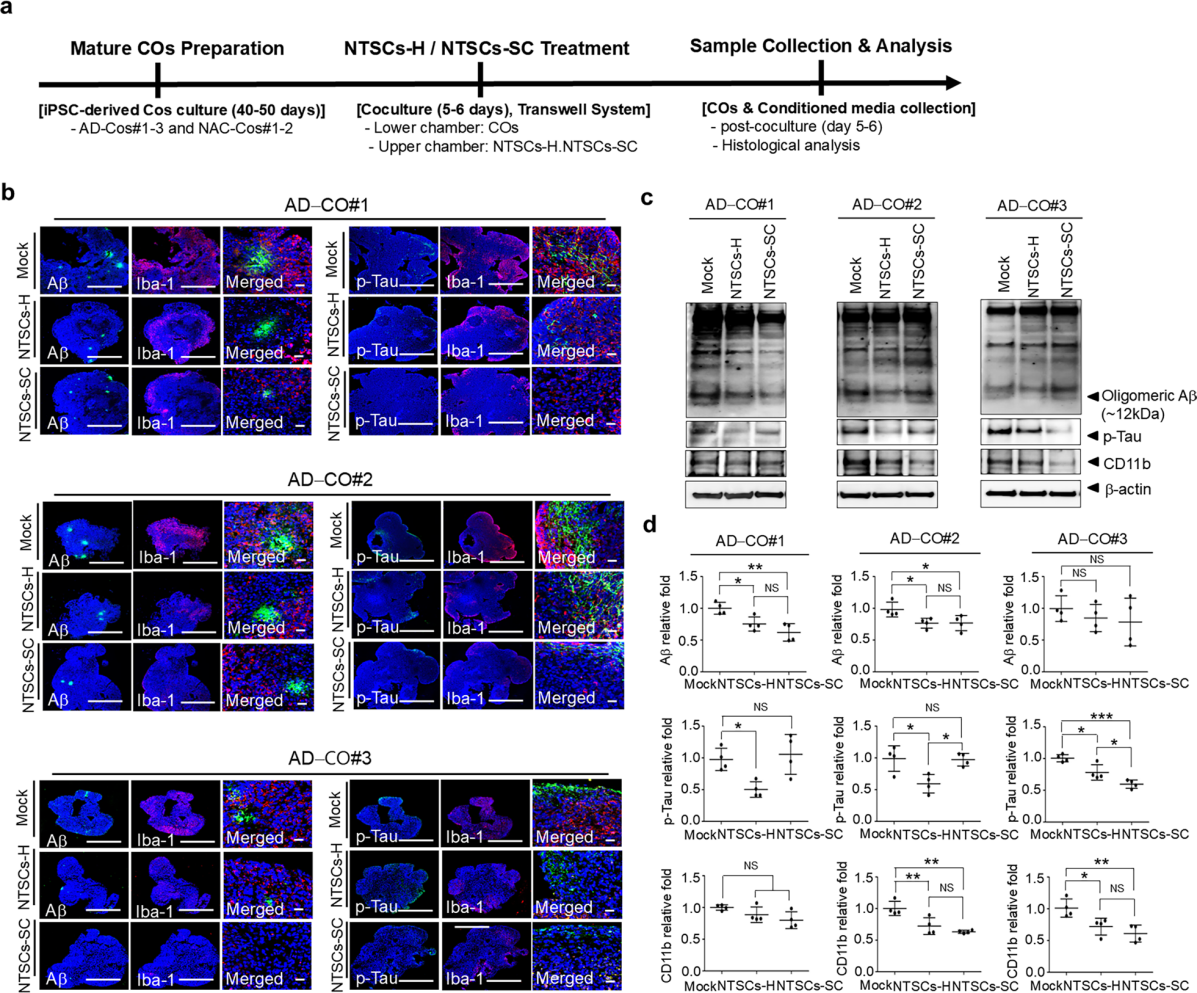
Effect of NTSCs-H or NTSCs-SC on AD-related pathological changes in AD-COs#1–3. **a** Schematic overview of coculture experiment. **b** Confocal microscopy images of paraffin-embedded sections of AD-COs#1–3 cocultured with either NTSCs-H or NTSCs-SC for 4 − 5 days. The sections were stained with the antibody 6E10 (green), an anti-Iba-1 antibody (red), and an anti-p-Tau (Ser202/Thr205) antibody (green). Nuclei were labeled with DAPI (blue). Scale bars: 500 μm (left and middle) and 20 μm (right). **c** Western blot analysis of AD-COs#1–3 cocultured with either NTSCs-H or NTSCs-SC for 5–6 days. **d** Quantification of the Western blot results. The values are the means (SDs). Statistical analysis was performed using one-way ANOVA followed by Tukey’s post hoc test, *P < 0.05, **P < 0.01, ***P < 0.001. All images and data are representative of two or three independent experiments

The ability of NTSCs-H to modulate AD-related pathological changes in comparison to NTSCs-SC, was assessed by culturing AD-COs#1–3 with either NTSCs-H or NTSCs-SC for 5–6 days followed by immunohistochemical staining (Fig. 6a). Confocal microscopy images showed clustering of microglia (Iba-1^+^) around Aβ aggregates (6E10) in untreated AD-COs#1–3 (Fig. 6b). The size and the number of Aβ aggregates were noticeably reduced in AD-COs#1–3 cocultured with NTSCs-H and NTSCs-SC (Fig. 6b). In addition, there was a high abundance of p-tau (Ser202/Thr205)-positive cells in mock-treated AD-COs#1–3. NTSCs-H markedly reduced the number of p-tau-positive cells, and NTSCs-SC demonstrated a similar therapeutic effect (Fig. 6b).

Western blot analysis using the 6E10 antibody detected multiple Aβ-specific bands in AD-COs#1–3. The levels of these Aβ isoforms were significantly reduced following coculture with either NTSCs-H or NTSCs-SC, despite individual variations among AD-COs#1–3 (Fig. 6c, d). The protein levels of p-tau and CD11b were also decreased in AD-COs#1–3 cocultured with NTSCs-H compared to mock-treated AD-COs#1–3 (Fig. 6c, d). However, no significant differences in AD-related protein levels were observed between AD-COs#1–3 cocultured with NTSCs-H and those cocultured with NTSCs-SC. These findings demonstrate that treatment with NTSCs-H, which contain a higher percentage of Muse cells, is equally effective as NTSCs-SC in alleviating AD-related pathology.

### NTSCs-H or NTSCs-SC increase the expression levels of neuronal markers in AD-COs#1–3

**Fig. 7:**
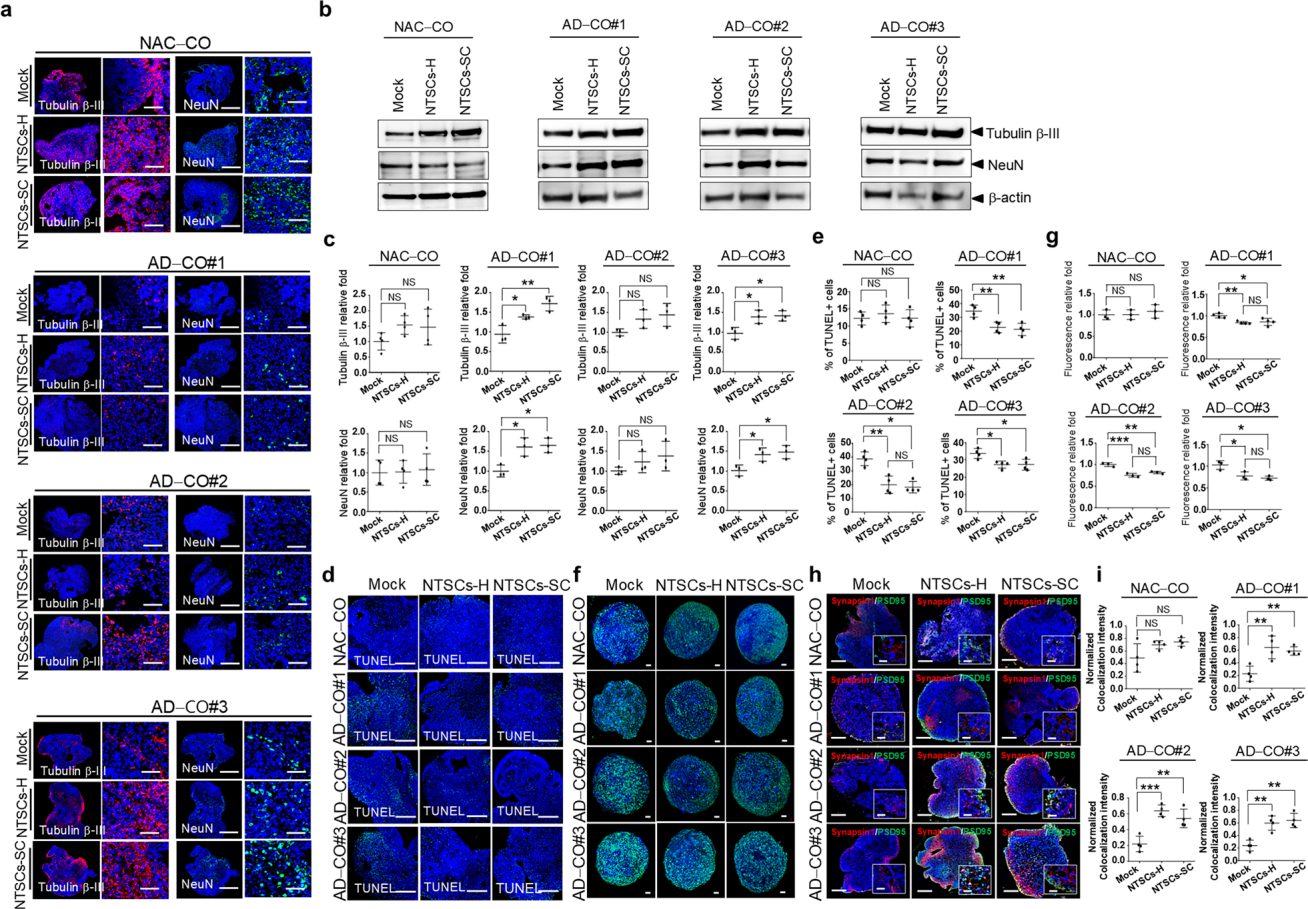
Effects of NTSCs-H or NTSCs-SC on the expression of neuronal cell marker in AD-COs#1–3. **a** Confocal microscopy images of paraffin-embedded sections of NAC-CO and AD-COs#1–3 cocultured with either NTSCs-H or NTSCs-SC for 5–6 days. The sections were double staining with antibodies against tubulin β − III (red) and NeuN (green). Nuclei were labeled with DAPI (blue). Scale bars: 500 μm (overview) and 50 μm (magnified). **b** Western blot analysis of NAC-CO and AD-COs#1–3 cocultured with either NTSCs-H or NTSCs-SC for 5–6 days. **c** Quantification of the Western blot results. The values are the means (SDs). **d** TUNEL staining of paraffin-embedded sections of NAC-CO and AD-COs#1–3 cocultured with either NTSCs-H or NTSCs-SC for 5–6 days. Nuclei were labeled with DAPI (blue). Scale bars: 200 μm. **e** Quantification of TUNEL-positive cells (*n* = 4 per group). **f** Fluo-4 staining in NAC-CO and AD-COs#1–3 cocultured with either NTSCs-H or NTSCs-SC for 5–6 days. Nuclei were labeled with DAPI (blue). Scale bar: 100 μm. **g** Quantification of fluorescence intensity is presented as the means (SDs). **h** Synapsin I (red) and PSD-95 (green) staining in paraffin-embedded sections of NAC-CO and AD-COs#1–3 cocultured with either NTSCs-H or NTSCs-SC for 5–6 days. Nuclei were labeled with DAPI (blue). Scale bars: 100 μm; inset, 20 μm. **i** Normalized colocalization intensity of Synapsin I and PSD-95. Data are presented as the means (SDs). One-way ANOVA, *P < 0.05, **P < 0.01, and ***P < 0.001

Following coculture with either NTSCs-H or NTSCs-SC for 5–6 days, immunohistochemical staining was performed for neuronal cell markers β-III tubulin and NeuN. Immunofluorescence staining revealed presence of neuronal cells in both NAC-CO#2 (NAC-CO) and AD-COs#1–3. Confocal microscopy demonstrated that coculture with either NTSCs-H or NTSCs-SC led to increased expression of the neuronal progenitor cell marker β-III tubulin and the mature neuronal marker NeuN in both NAC-CO and AD-COs#1–3 (Fig. 7a). Western blot analysis further confirmed increased levels of β-III tubulin and NeuN in AD-COs#1–3 cocultured with NTSCs-H or NTSCs-SC compared to mock-treated AD-COs#1–3. However, due to the heterogeneity of AD-related pathological changes across AD-COs#1–3, the relative expression of β-III tubulin and NeuN, normalized to β-actin, did not show consistent changes (Fig. 7b, c). Additionally, no significant differences in the levels of neuronal markers were observed between AD-COs#1–3 cocultured with NTSCs-H and those cocultured with NTSCs-SC. In NAC-CO, the β-III tubulin level increased approximately 1.5-fold following coculture with either NTSCs-H or NTSCs-SC, with no significant difference between the two conditions. TUNEL staining revealed a significant increase in TUNEL-positive cells in AD-COs#1–3 compared to NAC-CO. NTSCs-H or NTSCs-SC significantly reduced the proportion of TUNEL-positive cells in AD-COs#1–3, while no such reduction was observed in NAC-CO (Fig. 7d, e).

To further assess neuronal function, intracellular calcium levels were examined, as calcium (Ca^2^⁺) homeostasis is critical for synaptic transmission, plasticity, and overall neuronal signaling [64]. In AD, dysregulated Ca^2^⁺ regulation and excessive Ca^2^⁺ influx contribute to neuroinflammation, oxidative stress, mitochondrial dysfunction, and neuronal apoptosis [65, 66]. Confocal imaging of organoids stained with the Ca^2^⁺-sensitive fluorescent dye Fluo-4 revealed elevated intracellular calcium levels in AD-COs compared with NAC-CO, indicating impaired calcium regulation. Notably, coculture with either NTSCs-H or NTSCs-SC significantly reduced Fluo-4 fluorescence intensity in AD-COs#1–3 compared with mock-treated AD-COs#1–3 (Fig. 7f, g), whereas no such reduction was observed in NAC-CO, suggesting restoration of calcium homeostasis specific to the AD condition.

To further evaluate dynamic neuronal activity, time-lapse calcium imaging was performed to measure Ca^2^⁺ oscillations in NAD-CO and AD-CO#1, as well as in those cocultured with either NTSCs-H or NTSCs-SC. Representative raw imaging videos used in this analysis are provided in supplementary material as Videos S1–S6. Distinct differences in spontaneous Ca^2^⁺ oscillatory activity were observed among groups. The mock-treated NAC-CO exhibited frequent, regular Ca^2^⁺ oscillations with consistent amplitudes. In contrast, the mock-treated AD-CO#1 displayed markedly diminished oscillatory activity, characterized by reduced frequency and amplitude of calcium transients. Remarkably, coculture with either NTSCs-H or NTSCs-SC restored Ca^2^⁺ oscillation frequency and amplitude to levels comparable to those observed in mock-treated NAC-CO, demonstrating recovery of functional calcium signaling and neuronal network activity (Fig. S3). These results indicate that calcium signaling is impaired in AD-COs and that this dysfunction can be effectively rescued by NTSCs-H or NTSCs-SC treatment.

To further investigate the impact of NTSCs-H on synaptic function, immunostaining was performed for the presynaptic and postsynaptic adaptor proteins Synapsin I and PSD-95 after 5–6 days of coculture. Confocal microscopy revealed that coculture with either NTSCs-H or NTSCs-SC increased the fluorescence intensity of both Synapsin I and PSD-95 in AD-COs#1–3 compared with mock-treated AD-COs#1–3 (Fig. 7h). Moreover, the colocalization of Synapsin I and PSD-95 was significantly enhanced with NTSCs-H or NTSCs-SC treatment relative to mock treatment, whereas no such changes were observed in NAC-CO (Fig. 7i). In addition, no significant differences in colocalization levels were detected between NTSCs-H and NTSCs-SC treatment. Together, these results demonstrate that both NTSCs-H and NTSCs-SC restore calcium signaling and promote synaptic connectivity, thereby improving neuronal function in AD organoids.

### Treatment with NTSCs-H or NTSCs-SC downregulates the expression of AD-related cytokines in AD-COs#1–3

**Fig. 8:**
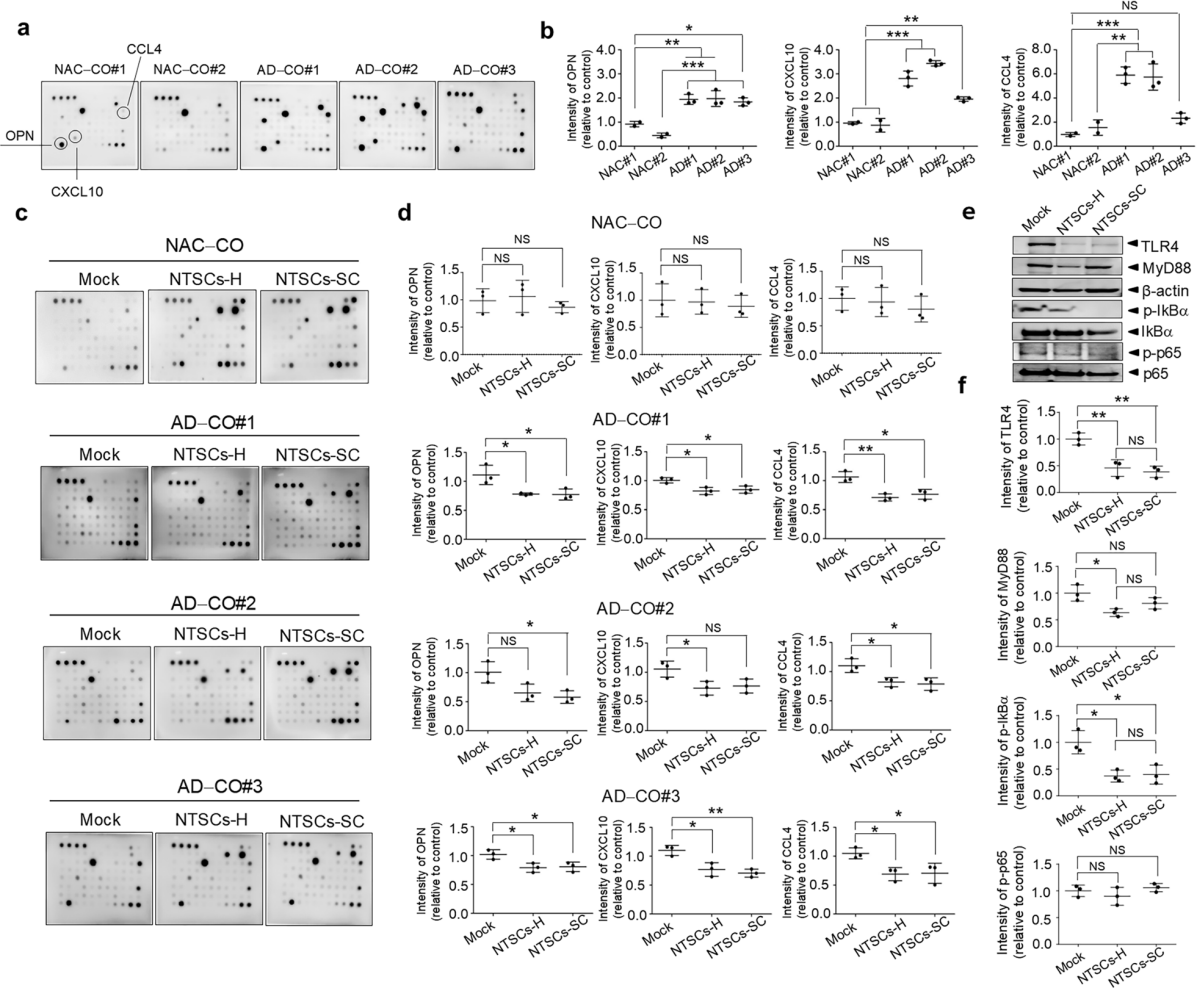
Effect of NTSCs-H or NTSCs-SC on the expression of AD-related cytokines in AD-COs#1–3. **a** Cytokine expression profiles obtained by incubating an array membrane with culture media from NAC-COs#1–2 and AD-COs#1–3. **b** Gray values of the OPN, CXCL10, and CCL4 spots on the array membrane. The values shown are the means (SDs). **c** Cytokine expression profiles obtained by incubating an array membrane with culture media from NAC-CO and AD-COs#1–3 cocultured with either NTSCs-H or NTSCs-SC for 5–6 days. **d** Gray values of the OPN, CXCL10, and CCL4 spots on the array membrane. The values shown are the means (SDs). **e** Western blot analysis of AD-COs#2 cocultured with either NTSCs-H or NTSCs-SC for 5–6 days. **f** Quantification of protein levels. All images and data are representative of two or three independent experiments. One-way ANOVA, *P < 0.05, **P < 0.01, and ***P < 0.001

Peripheral levels of various inflammation-related cytokines and chemokines are altered during AD pathogenesis and are strongly associated with disease progression [67, 68]. Quantification of cytokines in media conditioned by NAC-COs#1–2 and AD-COs#1–3 using a human cytokine array kit revealed that the levels of OPN and CXCL10 were approximately 2.0–4.0-fold higher, and CCL4 approximately 1.5–5.0-fold higher, in AD-COs#1–3 compared to NAC-COs#1–2 (Fig. 8a, b).

However, the levels of CXCL10 and CCL4 were reduced by approximately 20%–50%, and OPN reduced by approximately 30%–50% in AD-COs#1–3 cocultured with either NTSCs-H or NTSCs-SC compared to mock-treated AD-COs#1–3. In contrast, no such reduction was observed in NAC-COs (Fig. 8c, d). Moreover, there were no significant differences in cytokine or chemokine levels between AD-COs#1–3 cocultured with NTSC-H and those with NTSC-SC.

To further elucidate the underlying mechanisms, we analyzed the expression levels of proteins involved in the NF-κB signaling pathway, a central regulator of inflammation that drives the transcription of various pro-inflammatory genes, including those encoding cytokines and chemokines [48, 49]. Western blot analysis revealed that the protein levels of TLR4, Myd88, and the phosphorylation levels of IkB, key upstream regulators of NF-κB signaling, were significantly reduced in AD-COs#2 cocultured with either NTSCs-H or NTSCs-SC compared to mock-treated AD-COs#2 (Fig. 8e, f). However, the phosphorylation level of NF-κB p65 was not decreased in either NTSCs-H- or NTSCs-SC-treated AD-COs#2. Furthermore, no significant differences were observed between the NTSCs-H and NTSCs-SC treatment groups (Fig. 8e, f). These findings suggest that treatment with either NTSCs-H or NTSCs-SC attenuates the activation of upstream NF-kB signaling and reduces the secretion of cytokines and chemokines associated with AD pathogenesis in COs derived from iPSCs of AD patients.

### Safety of NTSCs in an in vivo model

**Fig. 9:**
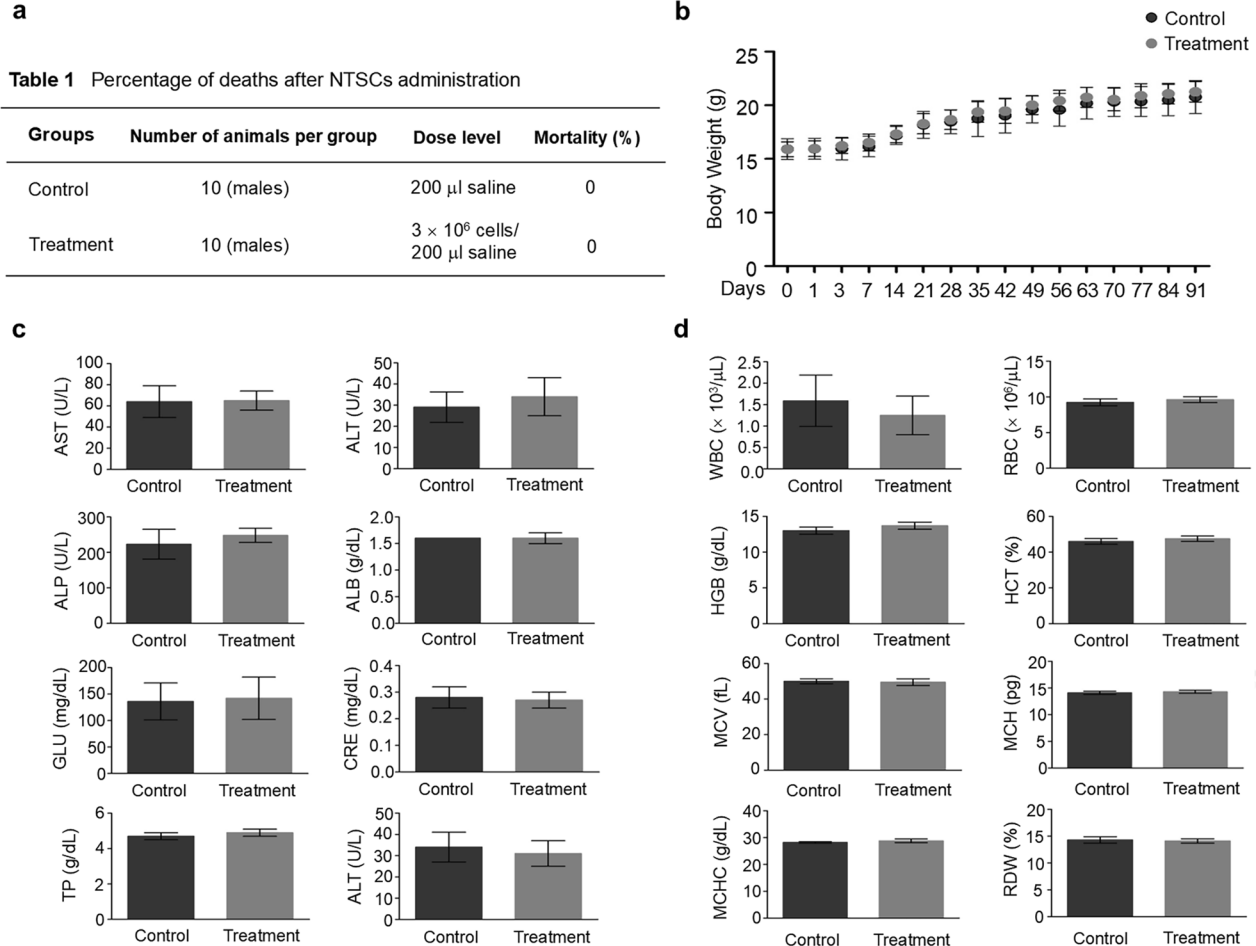
Analysis of the in vivo toxicity of NTSCs. **a** Percentage of death by 13 weeks after intravenous injection of NTSCs from a healthy donor (3 × 10^6^ cells/mouse, *n* = 10 per group). **b** Body weights of mice that received saline (control group) or NTSCs (treatment group). **c, d** Biochemical and hematological parameters in mice that received saline (control group) or NTSCs (treatment group). The values shown are the means (SDs). Student’s *t*-test

Following the determination that Muse cells (SSEA3^+^/CD105^+^) can serve as an effective marker for predicting the therapeutic efficacy of NTSCs in treating AD, an in vivo toxicity study was conducted to evaluate the safety of NTSCs. A total of 3 × 10^6^ NTSCs were injected intravenously to each WT mouse, while saline was injected as a control. All mice in the NTSC treatment group survived until the designated endpoint for toxicity analysis (Fig. 9a). No significant difference in body weight was observed between the control and treatment groups during the 13-week observation period (Fig. 9b). Liver function tests, an essential component of toxicity assessment, revealed no statistically significant differences in serum levels of liver function markers, including aspartate aminotransferase (AST), alanine aminotransferase (ALT), alkaline phosphatase (ALP), albumin (ALB), glucose (GLU), creatinine (CRE), and total protein (TP), between the two groups at 13 weeks post-injection (Fig. 9c). Similarly, complete blood count parameters including white blood cell (WBC) count, red blood cell (RBC) count, hemoglobin (HGB) level, hematocrit (HCT), mean corpuscular volume (MCV), mean corpuscular hemoglobin (MCH), mean corpuscular hemoglobin concentration (MCHC), and RBC distribution width (RDW) did not differ significantly between the two groups (Fig. 9d). These findings demonstrate that NTSCs are safe in vivo in the animal model, supporting their feasibility as a therapeutic option for the treatment of AD.

## Discussion

In this study, we investigated the potential of Muse cells (SSEA3^+^/CD105^+^) as a marker for predicting the therapeutic efficacy of NTSCs in treating AD. NTSCs derived from various donors alleviated cognitive impairment and AD-associated neuropathological changes to varying degrees in 5 × FAD Tg AD model mice, potentially depending on the percentage of Muse cells (SSEA3^+^/CD105^+^) within the NTSCs. NTSCs with the highest percentage of Muse cells (SSEA3^+^/CD105^+^) showed the greatest efficacy in improving AD-related pathology and cognitive deficits. Compared with NTSCs with a lower percentage of SSEA3^+^/CD105^+^ cells (NTSC-L), NTSCs with a higher percentage of SSEA3^+^/CD105^+^ cells (NTSCs-H) showed a greater proliferative capacity, multilineage differentiation potency, and secretion of neuroprotective cytokines that were comparable to pure SSEA3^+^/CD105^+^ cells isolated from NTSCs (NTSC-SC) in culture. Both NTSCs-H and NTSCs-SC improved cognitive deficits and reduced cerebral Aβ deposition, inflammation, and neuronal death in AD model mice. Furthermore, NTSCs-H and NTSCs-SC mitigated AD pathology in AD COs by reducing Aβ aggregation, tau hyperphosphorylation, astrogliosis, neuronal death, and inflammatory cytokine levels. Collectively, these findings indicate that NTSCs-H and NTSCs-SC exhibit comparable therapeutic potentials for the treatment of AD. Although inter-donor variability has been an issue resulting in unpredictable clinical outcomes of stem cell therapy, in the present study we verified that NTSCs-H, which contained high percentage of Muse cells (SSEA3^+^/CD105^+^), can rapidly improve cognitive function and greatly mitigate pathological changes associated with AD, supporting that Muse cells (SSEA3^+^/CD105^+^) are potential quality markers for determining the efficacy of NTSCs in treating AD.

Human neural crest-derived NTSCs show good potential for treating AD and can be isolated from the nasal turbinate; these stem cells are easily accessible and can be obtained through minimally invasive procedures during turbinate resection [25, 26]. NTSCs can potentially differentiate into neurons [27], making them suitable candidates for the treatment of neurodegenerative diseases such as AD and Parkinson’s disease. Moreover, NTSCs possess immunomodulatory properties [69], allowing them to reduce inflammation and modulate immune responses in diseases involving inflammation. Recently, NTSC therapy was shown to regulate microglial activation and ameliorate AD-related neuropathological changes in the AD brain [27]. In the present study, NTSCs were demonstrated to be safe, with no signs of toxicity, highlighting their potential as a promising stem cell-based therapy for patients with AD. Furthermore, in our previous study, SSEA3⁺/CD105⁺ NTSCs were transplanted into immunodeficient NOD/SCID-γ (NSG) mice and monitored for up to 329 days without observation of any evidence of tumor formation [70]. These findings further support the long-term safety and non-tumorigenic nature of NTSCs. However, more comprehensive investigations, including long-term viability, immune tolerance, molecular biomarker profiling (oncogene activation, cytokine storm markers), and safety assessments in immunocompetent hosts, are necessary to validate their clinical applicability. Addressing these factors will be crucial for successful translation of NTSC-based therapies into clinical settings.

In this study, we validated the variation in the ability of NTSCs from different donors to treat AD. Aβ accumulation is a central feature of AD pathology and is closely linked to cognitive impairment [1, 2]. Aβ plaques are neurotoxic and disrupt normal brain function. They interfere with synaptic function, impair neuronal communication, and induce inflammation and oxidative stress [71]. These effects contribute to the progressive degeneration and loss of neurons in brain regions critical for memory and cognition. NTSCs derived from various donors improved cognitive impairment and AD-associated neuropathological changes to varying degrees in 5 × FAD Tg AD model mice, depending on the percentage of Muse cells (SSEA3^+^/CD105^+^). NTSCs containing the highest percentage of Muse cells (SSEA3^+^/CD105^+^) showed the greatest efficacy in improving AD-related pathology and cognitive deficits.

Muse cells can differentiate into cells of all three germ layers. Muse cells have been demonstrated to exhibit immunomodulatory properties in a wide range of disorders including stroke [72, 73], amyotrophic lateral sclerosis (ALS) [74, 75], and myocardial infarction (MI) [76]. They also exert anti-inflammatory, anti-apoptotic, and tissue-protective effects [29, 31]. Based on the promising findings of preclinical studies, Muse cells have been used for the treatment of patients with stroke, SCI, and MI in clinical trials [77, 78]*.* Although Muse cells (SSEA3^+^/CD105^+^) can be derived from various sources and exhibit strong therapeutic potential, their low abundance makes it difficult to obtain sufficient numbers for clinical application.

In this study, compared with NTSCs-L, NTSCs-H and NTSCs-SC showed greater proliferative capacity, multiple differentiation potency, and secretion of neuroprotective cytokines in culture, with no significant differences observed between the two, further suggesting that NTSCs-H have comparable in vitro stem cell properties to NTSCs-SC.

Moreover, both NTSCs-H and NTSCs-SC improved cognitive deficits and reduced cerebral Aβ deposition, inflammation, and neuronal death in AD model mice, with no significant difference between NTSCs-H and NTSCs-SC. These findings suggest that both NTSCs-H and NTSCs-SC exhibit comparable therapeutic potential for the treatment of AD. These data reveal that NTSCs-H that contain a high percentage of Muse cells (SSEA3^+^/CD105^+^) are valuable cells for the treatment of AD due to their great stem cell properties in vitro and therapeutic potency in vivo to improve AD-related cognitive impairment and mitigate neuropathological changes.

Our in vivo findings demonstrate that transplantation of NTSCs-H and NTSCs-SC significantly reduced microglial activation, decreased levels of proinflammatory cytokines (IL-6, TNF-α, IL-1β, and OPN), and attenuated Aβ pathology, accompanied by improved cognitive function in 5 × FAD AD model mice. These neuroimmune changes were associated with downregulation of the TLR4/MyD88/NF-κB signaling components. Although these results indicate a modulatory role of NTSCs on neuroinflammation, the precise mechanisms, whether through paracrine signaling, cell–cell interactions, or indirect bystander effects, remain to be fully defined. The elevated expression of neurotrophic and immunomodulatory cytokines such as TIMP2, VEGF, BDNF, and GDNF in NTSCs suggests that paracrine signaling may be a key contributor. However, further mechanistic studies are necessary to clarify the interactions between NTSCs and microglia in the AD brain environment.

While our findings support the therapeutic potential of NTSCs in modulating the pathological environment of AD, the precise mechanisms underlying these effects remain to be fully elucidated. Although a small subset of transplanted cells expressed neuronal markers, our findings suggest that paracrine signaling, rather than direct neuronal replacement, is likely the primary mechanism underlying the observed therapeutic benefits. Notably, NTSCs with greater therapeutic efficacy exhibited higher expression levels of neurotrophic and immunomodulatory factors. However, the direct causal roles of these cytokines in the therapeutic effects remain to be validated. Future studies are essential to clarify the relative contributions of secreted factors, cell–cell interactions, and cellular differentiation to the effects of NTSCs in AD.

It is important that NTSCs-H and NTSCs-SC have equivalent effects in improving cognitive impairment and mitigating pathological changes associated with AD. Muse cell (SSEA3^+^/CD105^+^) isolation and expansion to achieve adequate numbers for clinical application is time-consuming and expensive. Therefore, it would be more beneficial to select and use NTSCs with a high proportion of Muse cells (SSEA3^+^/CD105^+^) for AD treatment. In this study, although Muse cells were initially purified to nearly 100%, we observed a progressive decline in their proportion over repeated passages. This phenomenon is consistent with previous findings by Kuroda et al. [28], which reported that Muse cells undergo asymmetric division under adherent culture conditions, generating both Muse and non-Muse progeny. Over time, the more rapidly proliferating non-Muse cells tend to dominate the culture, leading to a gradual decrease in the overall Muse cell content. In the present study, NTSCs-SC (initially purified Muse cells) were expanded in parallel with NTSCs-H up to passage 5 for use in both in vivo and in vitro AD models. Flow cytometry and immunofluorescence analyses revealed that the Muse cell proportion had declined to approximately 9.3% by passage 5, the exact population used in our functional assays (Fig. S4). In contrast, NTSCs-H at the same passage displayed a Muse cell content of approximately 16.9%. This convergence in Muse cell proportions between NTSCs-SC and NTSCs-H at the time of administration likely accounted for the comparable therapeutic outcomes observed between the two groups. Moreover, it is plausible that non-Muse cells contributed to the therapeutic effects through paracrine mechanisms. Previous studies have shown that MSCs can exert immunomodulatory and regenerative effects by secreting a variety of bioactive factors and engaging in cell–cell interactions, which may support tissue repair and modulate immune responses [39, 41]. Therefore, the observed therapeutic efficacy may reflect both Muse-specific activity and auxiliary contributions from non-Muse cells within the NTSC population. Given the high cost and technical complexity of isolating and maintaining pure Muse cell populations, selecting NTSC populations with naturally enriched Muse cell content may be a more feasible and clinically translatable approach. To further optimize therapeutic outcomes, future studies should systematically investigate the dose–response relationship between Muse cell proportion and treatment efficacy, with the goal of establishing evidence-based thresholds for clinical application.

Extending these promising findings, we validated the feasibility of NTSCs-H as a promising cell type for treating AD in clinical settings with COs derived from iPSCs of AD patient. Animal models of AD do not fully reflect the neuropathology and disease progression observed in AD patients, and the differences between human and mouse brains significantly hinder translation from animal-based studies to humans [79]. COs, which can be derived from iPSCs due to their self-organizing ability, mimic the organized structure of the human brain while exhibiting key brain developmental processes [79, 80]. COs derived from the iPSCs of familial or sporadic AD patients exhibit robust AD-related pathological changes [81–83], supporting their use for validating the efficacy of potential therapeutic options for patients with AD. Our data showed that COs generated from the iPSCs of three sporadic AD patients (AD-COs#1, AD-COs#2, and AD-COs#3) displayed key AD-related pathological features, including Aβ aggregation, tau phosphorylation, inflammation, cell apoptosis and astrogliosis. In contrast, these factors were not observed in COs generated from the iPSCs of two healthy controls (NAC-COs#1 and NAC-COs#2). The pathological changes of COs derived from AD patient iPSCs showed inter-individual variability. Moreover, integrated scRNA-seq analysis revealed distinct molecular alterations in AD-COs, characterized by reduced Notch signaling in progenitor populations and an expansion of both immature and mature neurons, accompanied by a loss of retinal progenitors. These cellular changes were accompanied by robust expression of *VCAM1*, *TSPO*, and *CCL2*, with *S100B* showing selective enrichment in microglia. These genes are well-established mediators of neuroinflammation and have been consistently reported to be upregulated in the brains of AD patients relative to controls [59–63]. It is also important to acknowledge that the control donors were 0 year old, whereas the AD donors were 70–76 years old. Although aging-related molecular signatures are largely reset during the iPSC reprogramming, this substantial difference in donor ages should be considered when interpreting comparative analyses between control- and AD-derived cells [84]. Collectively, the recapitulation of key neuropathological features of AD in AD-COs highlights their potential as a valuable model system for investigating the therapeutic efficacy of NTSCs in the treatment of AD.

Although the iPSC-derived AD organoid model used in this study lacks certain in vivo features, such as a functional blood–brain barrier, systemic immune cells, and mature microglia, it successfully recapitulates key pathological hallmarks of AD. Furthermore, the organoids contained diverse neural and inflammatory cell populations, reflecting aspects of AD-relevant cellular complexity. Nonetheless, limitations such as the absence of peripheral immune components and vascularization, as well as inter-organoid heterogeneity, must be addressed. Future work should focus on refining organoid models and establishing standardized protocols to evaluate and minimize variability. These improvements will enhance the utility of AD organoids in disease modeling and preclinical therapeutic screening.

Notably, we observed that short-term (5–6 day) co-culture with NTSCs-H markedly mitigated AD-associated pathologies, including Aβ aggregation, tau hyperphosphorylation, neuronal cell death, and neuroinflammation, in AD-COs#1–3, whereas NTSCs-SC exerted comparable therapeutic effects. Moreover, NTSCs-H and NTSCs-SC both effectively inhibited progressive neuronal loss and improved neuronal integrity and function in AD-COs#1–3, with no significant differences between them. It should be noted, however, that Fluo-4 staining in this study provided a static representation of intracellular calcium levels rather than dynamic calcium transients; therefore, interpretations regarding neuronal excitability and functional activity should be made with caution. These findings indicate that NTSCs-H are as effective as NTSCs-SC in ameliorating AD-related pathology and protecting against neuronal loss in COs derived from AD patient iPSCs, despite individual variability among AD-COs#1–3. However, the long-term stability and functional relevance of these effects remain to be established. Future studies incorporating optimized long-term organoid culture systems will be essential to assess whether these molecular and cellular changes are sustained and whether they contribute to prolonged neuronal survival, organoid maturation, and durable therapeutic efficacy. Such investigations will help validate the translational relevance of NTSC-based interventions in AD.

It is important to establish standardized criteria and reliable methods for selecting NTSCs with high therapeutic efficacy for the treatment of AD. In this study, we found that Muse cell content may serve as a predictive indicator of NTSC efficacy in AD therapy. However, as with other MSC-based therapies, donor-to-donor variability remains a significant factor influencing therapeutic outcomes. Our findings revealed noticeable inter-donor variation in NTSC efficacy, underscoring the need for consistent and reproducible quality control measures in NTSC preparation. Therefore, the development of strategies to assess and ensure the quality and therapeutic potential of NTSCs, such as identifying key cellular characteristics or establishing potency assays, is crucial for improving clinical outcomes. In this study, Muse cells were isolated from the NTSC-H donor, which exhibited the highest therapeutic efficacy, but donor-specific effects cannot be excluded. Future studies directly comparing Muse cells from both high- and low-performing NTSC donors will be necessary to determine whether therapeutic benefits are primarily driven by Muse cell–intrinsic properties or influenced by donor variability. Such efforts will contribute to optimizing NTSC selection strategies, ensuring consistent product quality, and advancing NTSCs as a viable and standardized stem cell therapy for AD.

## Conclusion

This study demonstrates that Muse cells (SSEA3^+^/CD105^+^) serve as a potential predictive marker for the therapeutic efficacy of NTSCs in AD. NTSCs with a higher proportion of Muse cells consistently showed superior therapeutic outcomes across in vitro, in vivo, and cerebral organoid models, and exhibited efficacy comparable to isolated Muse cells. These findings indicate that SSEA3/CD105 positivity can be used as a practical criterion for selecting NTSCs with high therapeutic potential. Further studies are warranted to establish standardized thresholds for Muse cell content and to define the dose–response relationship between Muse cell proportion and treatment efficacy to support clinical translation.

## Supplementary Information

Additional file 1. **Fig. S1**. Quality control and clustering of integrated scRNA-seq data from AD-COs (#1–2) and NAC-COs (#1–2). **Fig. S2**. Characterization of AD-COs and NAC-COs by single-cell transcriptomic profiling. **Fig. S3**. Representative Ca^2+^ oscillation traces in NAC-CO and AD-CO#1 cocultured with either NTSCs-H or NTSCs-SC for 5–6 days. **Fig. S4**. Presence of Muse cells (SSEA3+/CD105+) populations in initially purified Muse cells from NTSCs and in cultured Muse cells (NTSCs-SC). **Table S5**. Summary of therapeutic effects according to Muse cell proportion in NTSCs.

Additional file 2. Detailed quantitative data.

Additional file 3. **Table S1.** Differentially expressed genes between AD-COs and NAC-Cos. **Table S2.** GSEA results of pathways enriched in AD-Cos.

Additional file 4. **Table S3.** Differential expression results in microglial cell type. **Table S4.** Differential expression results in astrocyte cell type.

Additional file 5. Representative raw imaging video for time-lapse calcium imaging to measure Ca^2+^ oscillations in AD-CO#1 Mock.

Additional file 6. Representative raw imaging video for time-lapse calcium imaging to measure Ca^2+^ oscillations in AD-CO#1_NTSCs-H.

Additional file 7. Representative raw imaging video for time-lapse calcium imaging to measure Ca^2+^ oscillations in AD-CO#1_NTSC-SC.

Additional file 8. Representative raw imaging video for time-lapse calcium imaging to measure Ca^2+^ oscillations in NAC-CO_Mock.

Additional file 9. Representative raw imaging video for time-lapse calcium imaging to measure Ca^2+^ oscillations in NAC-CO_NTSCs-H.

Additional file 10. Representative raw imaging video for time-lapse calcium imaging to measure Ca^2+^ oscillations in NAC-CO_NTSC_SC.

Additional file 11. Uncropped gels and blots.

## Data Availability

The datasets generated and/or analyzed during the current study are available from the corresponding author on reasonable request.

## Abbreviations

Aβ: Amyloid beta
AD: Alzheimer’s disease
BMSCs: Bone marrow-derived mesenchymal stem cells
COs: Cerebral organoids
FBS: Fetal bovine serum
HuNu: Human nuclei
iPSCs: Induced pluripotent stem cells
IL-1β: Interleukin-1β
IL-6: Interleukin-6
α-MEM: α-Mminimum essential medium
MSCs: Mesenchymal stem cells
Muse: Multilineage differentiating stress-enduring
Myd88: Myeloid differentiation primary response gene-88
NF-kB: Nuclear factor-κB
NTSCs: Neural crest-derived nasal turbinate stem cells
NEP: Neprilysin
OPN: Osteopontin
SSEA-3: Stage-specific embryonic antigen-3
TLR4: Toll-like receptor 4
TNF-α: Tumor necrosis factor-α
