# Supplementary material for: SSEA3 and CD105 positivity are associated with the treatment potency of human neural crest-derived nasal turbinate stem cells for Alzheimer’s disease
Source: Transl Neurodegener. 2026 Mar 3;15:8. doi: 10.1186/s40035-026-00539-3 (PMC12955099; doi:10.1186/s40035-026-00539-3)
Supplement: Supplementary file 1 — Additional file 1. Fig. S1. Quality control and clustering of integrated scRNA-seq data from AD-COs (#1–2) and NAC-COs (#1–2). Fig. S2. Characterization of AD-COs and NAC-COs by single-cell transcriptomic profiling. Fig. S3. Representative Ca2+ oscillation traces in NAC-CO and AD-CO#1 cocultured with either NTSCs-H or NTSCs-SC for 5–6 days. Fig. S4. Presence of Muse cells (SSEA3+/CD105+) populations in initially purified Muse cells from NTSCs and in cultured Muse cells (NTSCs-SC). Table S5. Summary of therapeutic effects according to Muse cell proportion in NTSCs. [file 40035_2026_539_MOESM1_ESM.docx]

SSEA3 and CD105 positivity are associated with the treatment potency of human neural crest-derived nasal turbinate stem cells for Alzheimer’s disease

Jung Yeon Lim^1†^, Jung Eun Lee^2†^, Minho Lee^3†^, Haewon Shim^3^, Sang In Park^4^, Soon A Park^5^, Sin−Soo Jeun^5^, Sheng*−*Min Wang^6^, Sunghwan Kim^6^, Seung Ho Yang^2*^, Hyun Kook Lim^6*^, Sung Won Kim^1*^

^1^Department of Otolaryngology-Head and Neck Surgery, Seoul St. Mary’s Hospital, College of Medicine, The Catholic University of Korea, Seoul, Republic of Korea

^2^Department of Neurosurgery, St. Vincent’s Hospital, College of Medicine, The Catholic University of Korea, Seoul, Republic of Korea

^3^Department of Life Science, Dongguk University, Seoul, Republic of Korea

^4^Institute of Catholic Integrative Medicine (ICIM), Incheon St. Mary's Hospital, The Catholic University of Korea, Incheon, Republic of Korea

^5^Department of Neurosurgery, Seoul St. Mary's Hospital, The Catholic University of Korea, Seoul, Republic of Korea

^6^Department of Psychiatry, Yeouido St. Mary’s Hospital, The Catholic University of Korea, Seoul, Republic of Korea

Correspondence: 72ysh@catholic.ac.kr (Seung Ho Yang); [drblues@catholic.ac.kr](mailto:drblues@catholic.ac.kr) (Hyun Kook Lim); kswent@catholic.ac.kr (Sung Won Kim)

Tel.: +82-2-2258-7535 ; Fax: +82-2-3482-1853

† These authors contributed equally to this work.


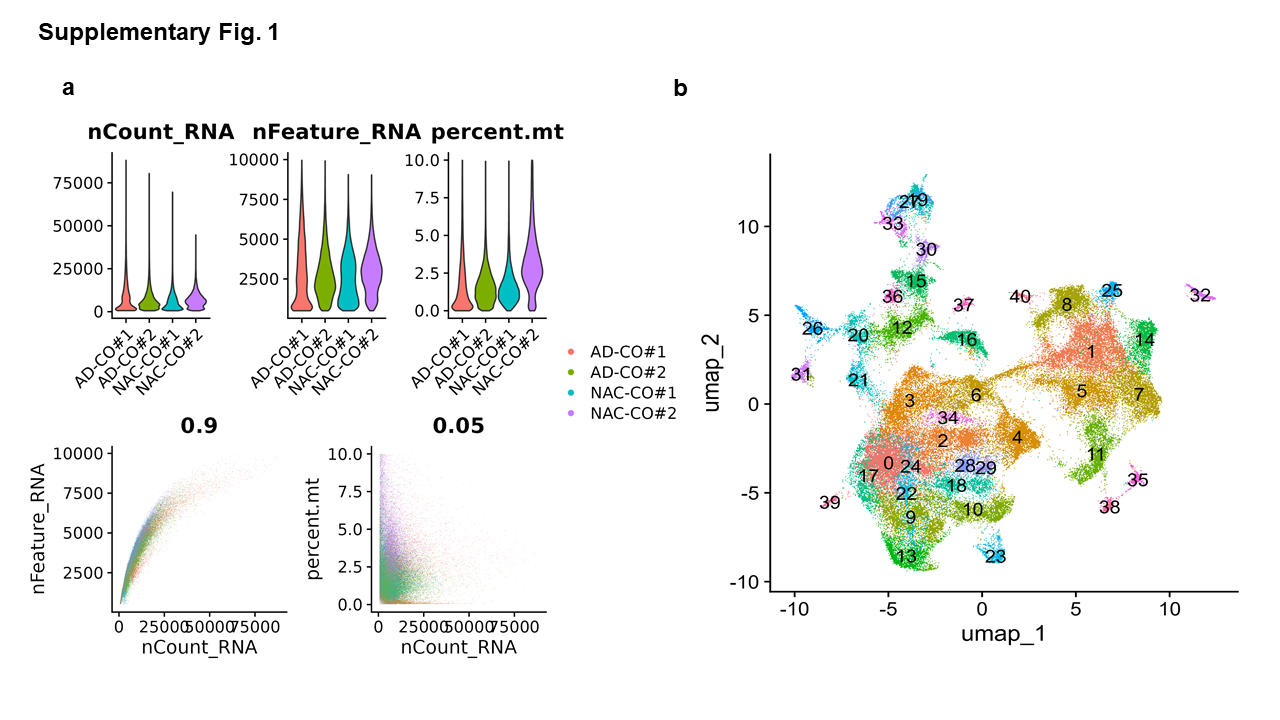


**Fig. S1. Quality control and clustering of integrated scRNA-seq data from AD-COs (#1–2) and NAC-COs (#1–2)**
**a** Quality control plots of scRNA-seq data from AD-COs and NAC-COs. Violin plots show the distributions of total UMI counts (nCount_RNA), the number of detected genes (nFeature_RNA), and the percentage of mitochondrial transcripts (percent.mt) for each sample. Scatter plots depict the relationships between UMI counts and the number of detected genes, as well as between UMI counts and mitochondrial gene percentages. Cells were filtered using the following thresholds: nCount_RNA > 1,500; 500 < nFeature_RNA < 10,000; and percent.mt < 10%.
**b** Uniform manifold approximation and projection (UMAP) plot of integrated scRNA-seq data from AD-COs and NAC-COs. The dataset was analyzed at a clustering resolution of 1.0, resulting in the identification of 41 distinct clusters.


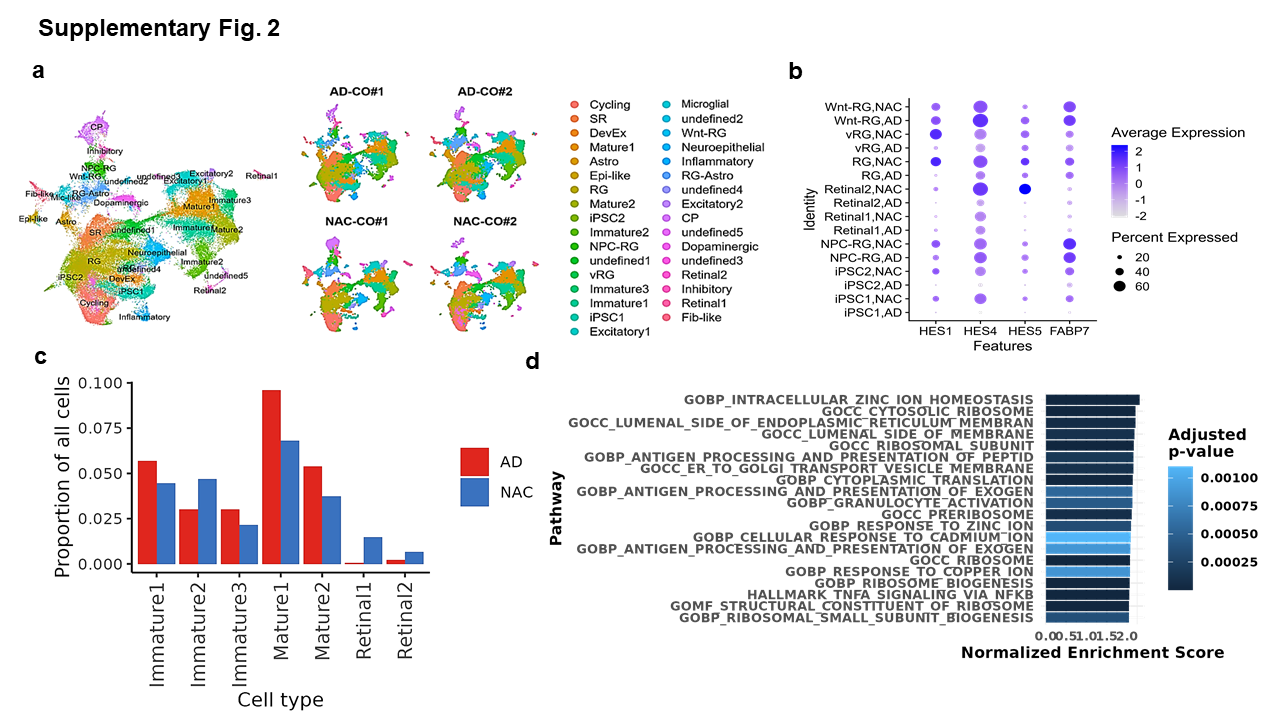


**Fig. S2. Characterization of AD-COs and NAC-COs by single-cell transcriptomic profiling**
**a** Uniform manifold approximation and projection (UMAP) plot showing integrated scRNA-seq data from 78,106 cells derived from AD-COs and NAC-COs. A total of 28 cell types were annotated. UMAP plots on the right display the scRNA-seq data for each individual CO.
**b** Dot plot showing the expression of Notch signaling genes across progenitor cell populations in AD-COs and NAC-COs. Color intensity indicates the average expression level of each gene, and dot size represents the proportion of cells expressing the gene within each cell type.
**c** Proportions of Immature, Mature, and Retinal cell types in AD-COs and NAC-COs. Bar plots show the relative frequencies of these cell types, revealing increased proportions of Immature and Mature neurons in AD-COs.
**d** Gene set enrichment analysis (GSEA) of differentially expressed genes between AD-COs and NAC-COs. The bar plot shows significantly enriched pathways ranked by normalized enrichment score (NES). Bar color represents the adjusted p-value (padj), and bar length reflects the NES for each pathway.


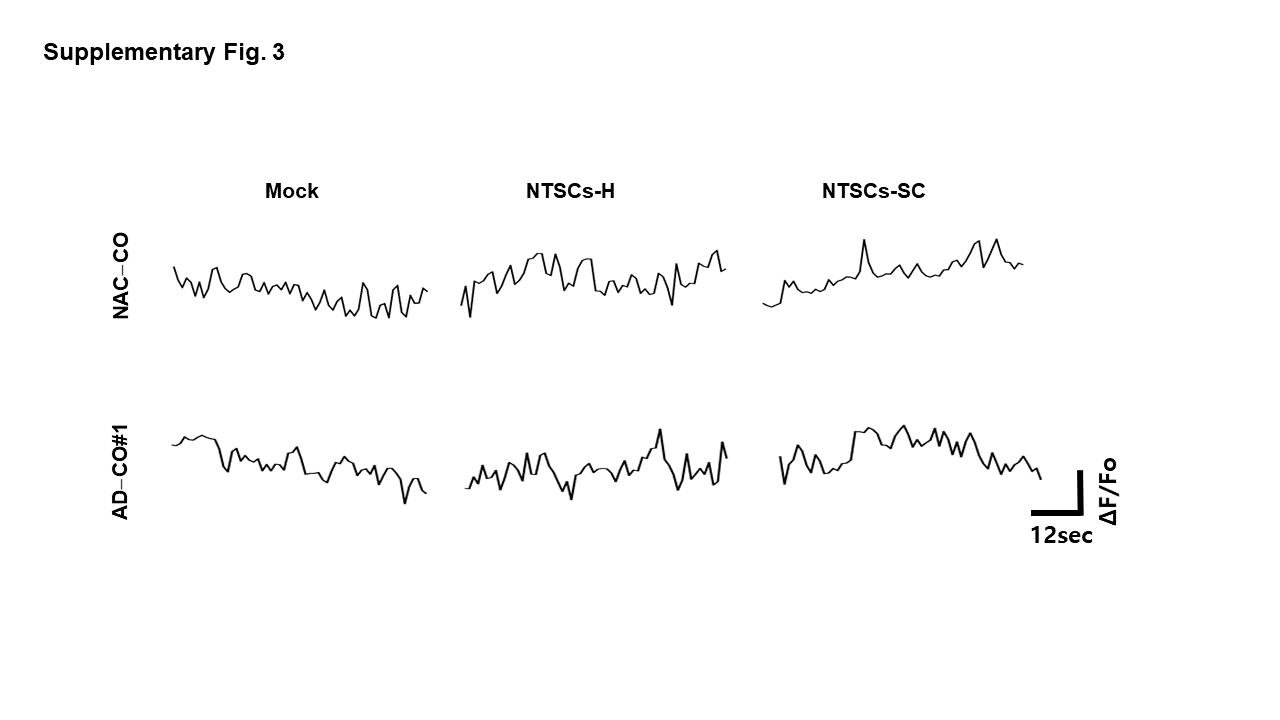


**Fig. S3.** **Representative Ca²⁺ oscillation traces in NAC-CO and AD-CO#1 cocultured with either NTSCs-H or NTSCs-SC for 5–6 days.**

Representative fluorescence intensity traces of the Ca²⁺-sensitive dye Fluo-4 recorded from selected regions of interest (ROIs) over time. Time-lapse calcium imaging was performed using an Olympus FV3000 confocal microscope under low-intensity illumination to minimize photobleaching. Fluorescence intensity F(t) was extracted from ROIs using Image J and normalized as ΔF/F₀ = (F(t) − F₀)/F₀. Representative Ca²⁺ traces are shown; the horizontal scale bar represents time (12 s), and the vertical scale bar indicates relative fluorescence changes (ΔF/F₀).

**
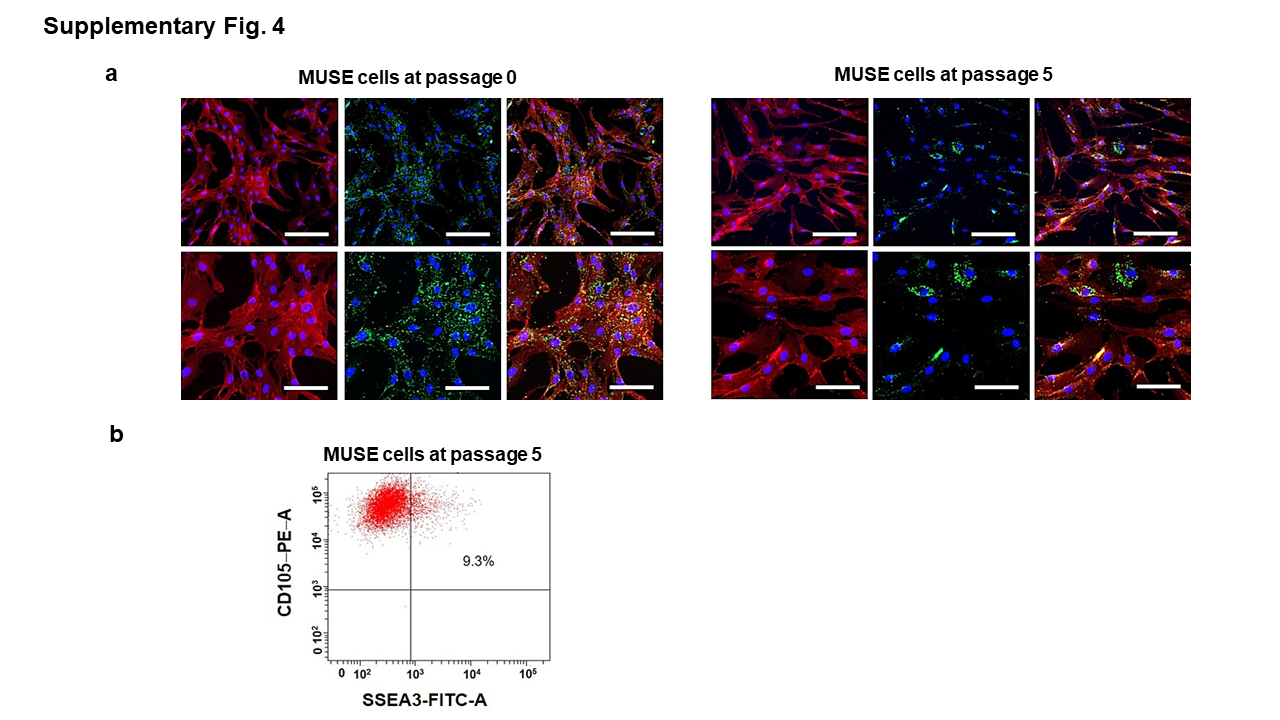
**

**Fig. S4. Presence of Muse cells (SSEA3+/CD105+) populations in initially purified Muse cells from NTSCs and in cultured Muse cells (NTSCs-SC).**

**a** Confocal microscopy images showing initially purified Muse cells derived from NTSCs (passage 0) and Muse cells expanded up to passage 5 in proliferation medium (passage 5), double-stained for SSEA3 (green) and CD105 (red). Scale bars: 100 μm and 200 μm. **b** Flow cytometry analyses of SSEA3 and CD105 expression in Muse cells at passage 5.

**
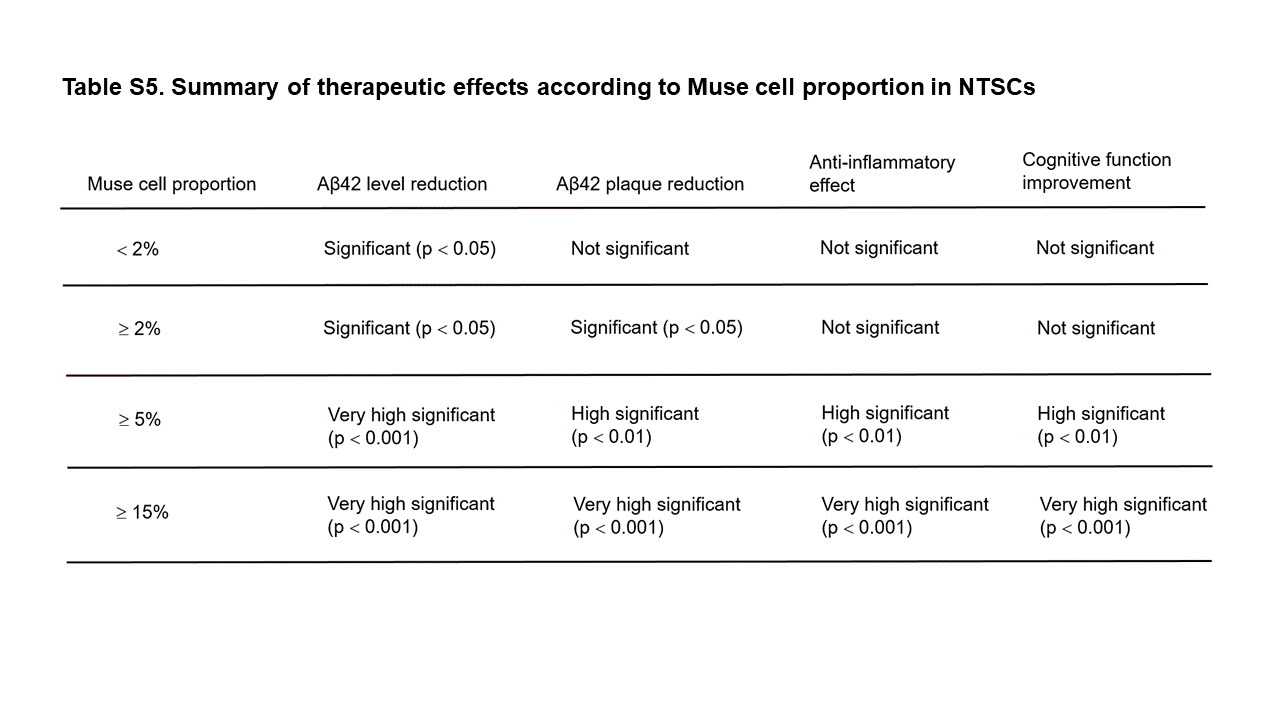
**
