# Supplementary material for: SSEA3 and CD105 positivity are associated with the treatment potency of human neural crest-derived nasal turbinate stem cells for Alzheimer’s disease
Source: Transl Neurodegener. 2026 Mar 3;15:8. doi: 10.1186/s40035-026-00539-3 (PMC12955099; doi:10.1186/s40035-026-00539-3)
Supplement: Supplementary file 2 — Additional file 2. Detailed quantitative data. [file 40035_2026_539_MOESM2_ESM.docx]

**Additional file 2. Detailed quantitative data**

Average escape latencies (mean ± SD) of WT-sham, Tg-sham, Tg-NTSC#1–5 groups in the Morris water maze test.

WT-sham: 21.9 ± 4.6 s

Tg-sham: 56.9 ± 6.0 s

Tg-NTSC#1: 32.7 ± 12.5 s

Tg-NTSC#2: 45.8 ± 16.7 s

Tg-NTSC#3: 47.9 ± 11.1 s

Tg-NTSC#4: 46.4 ± 13.3 s

Tg-NTSC#5: 27.9 ± 8.8 s

Quantitative analysis of Aβ plaque load (percentage of Aβ-positive area relative to total area) and the proportion of Iba-1–positive microglia in the brains of Tg-sham and Tg-NTSC#1–5 mice at 7 weeks post-transplantation. Data are presented as mean ± SD.

**Aβ plaque load (%)**

Tg-sham: 4.7 ± 1.1

Tg-NTSC#1: 2.1 ± 0.4

Tg-NTSC#2: 2.8 ± 0.7

Tg-NTSC#3: 2.7 ± 0.5

Tg-NTSC#4: 3.3 ± 0.3

Tg-NTSC#5: 1.4 ± 0.6

**Iba-1–positive cell percentages (%)**

Tg-sham: 9.8 ± 1.9

Tg-NTSC#1: 6.5 ± 1.0

Tg-NTSC#2: 7.2 ± 1.0

Tg-NTSC#3: 8.2 ± 0.4

Tg-NTSC#4: 8.1 ± 1.1

Tg-NTSC#5: 5.1 ± 0.7

Quantitative analysis of Aβ plaque load, expressed as the percentage of Aβ-positive area relative to the total area, in Tg-sham, Tg-NTSC-H, and Tg-NTSC-SC groups. Data are presented as mean ± SD.

Tg-sham: 6.6 ± 1.5%

Tg-NTSC-H: 1.8 ± 0.3%

Tg-NTSC-SC: 1.2 ± 0.3%

Average escape latencies (mean ± SD) of WT-sham, Tg-sham, Tg-NTSC-H, and Tg-NTSC-SC groups on day 7 of the Morris water maze training.

WT-sham: 14.8 ± 3.4 s

Tg-sham: 47.5 ± 10.5 s

Tg-NTSC-H: 26.4 ± 10.7 s

Tg-NTSC-SC: 22.3 ± 8.0 s

Time spent in the target quadrant (zone 4) during the probe test for WT-sham, Tg-sham, Tg-NTSC-H, and Tg-NTSC-SC groups. Data are presented as mean ± SD.

WT-sham: 39.6 ± 10.0 s

Tg-sham: 18.9 ± 14.6 s

Tg-NTSC-H: 39.6 ± 8.6 s

Tg-NTSC-SC: 42.8 ± 9.9 s

Quantification of TUNEL-positive cells in the cortical and hippocampal regions of Tg-sham, Tg-NTSC-H, and Tg-NTSC-SC groups at 7 weeks post-transplantation. Data are presented as mean ± SD.

Tg-sham: 28.0 ± 4.18 %

Tg-NTSC-H: 9.0 ± 2.9 %

Tg-NTSC-SC: 9.7 ± 2.4 %
