# Supplementary material for: SSEA3 and CD105 positivity are associated with the treatment potency of human neural crest-derived nasal turbinate stem cells for Alzheimer’s disease
Source: Transl Neurodegener. 2026 Mar 3;15:8. doi: 10.1186/s40035-026-00539-3 (PMC12955099; doi:10.1186/s40035-026-00539-3)
Supplement: Supplementary file 11 — Additional file 11. Uncropped gels and blots. [file 40035_2026_539_MOESM11_ESM.pptx]

## Slide 1
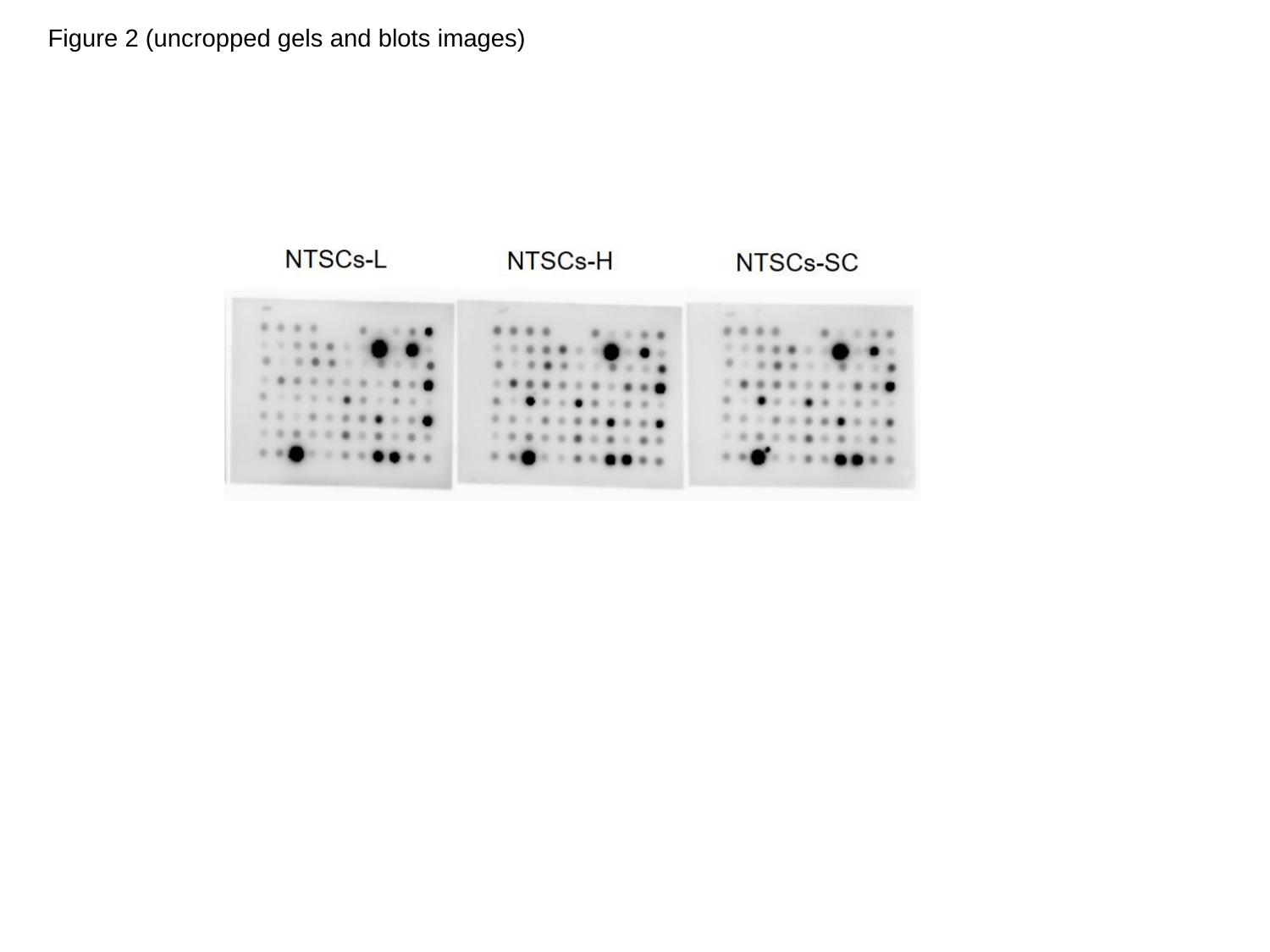

Figure 2 (uncropped gels and blots images)

## Slide 2
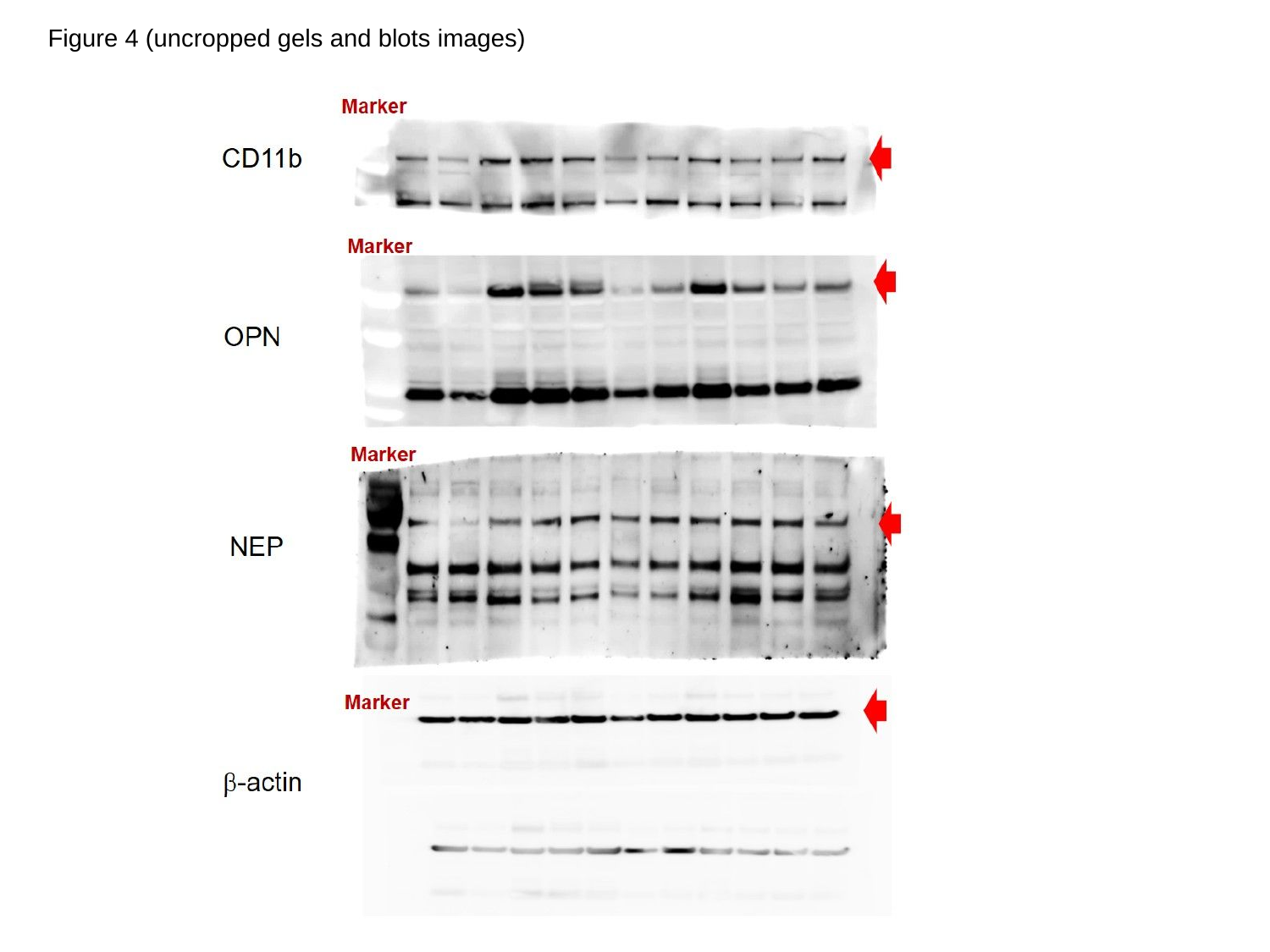

Figure 4 (uncropped gels and blots images)

## Slide 3
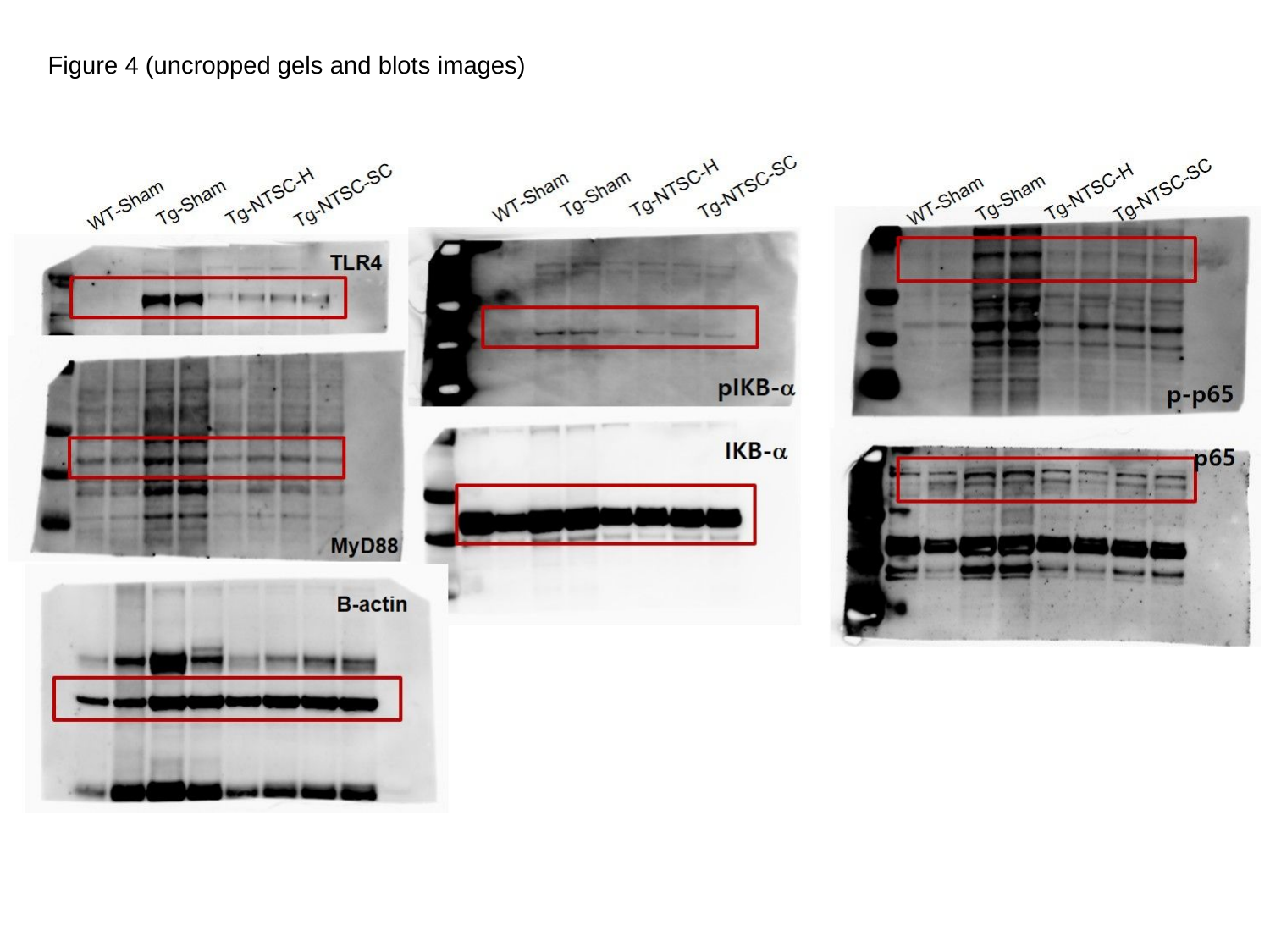

Figure 4 (uncropped gels and blots images)

## Slide 4
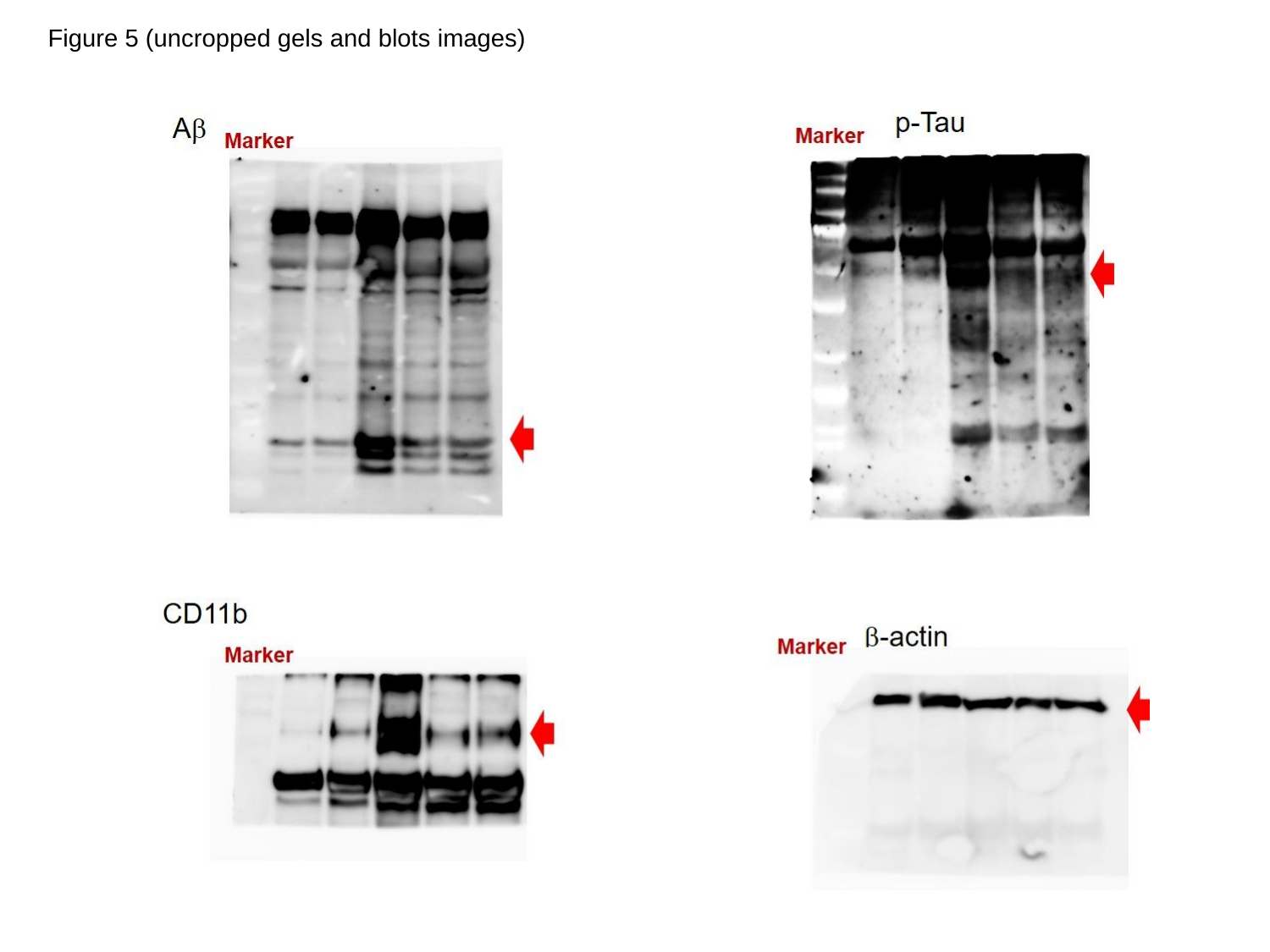

Figure 5 (uncropped gels and blots images)

## Slide 5
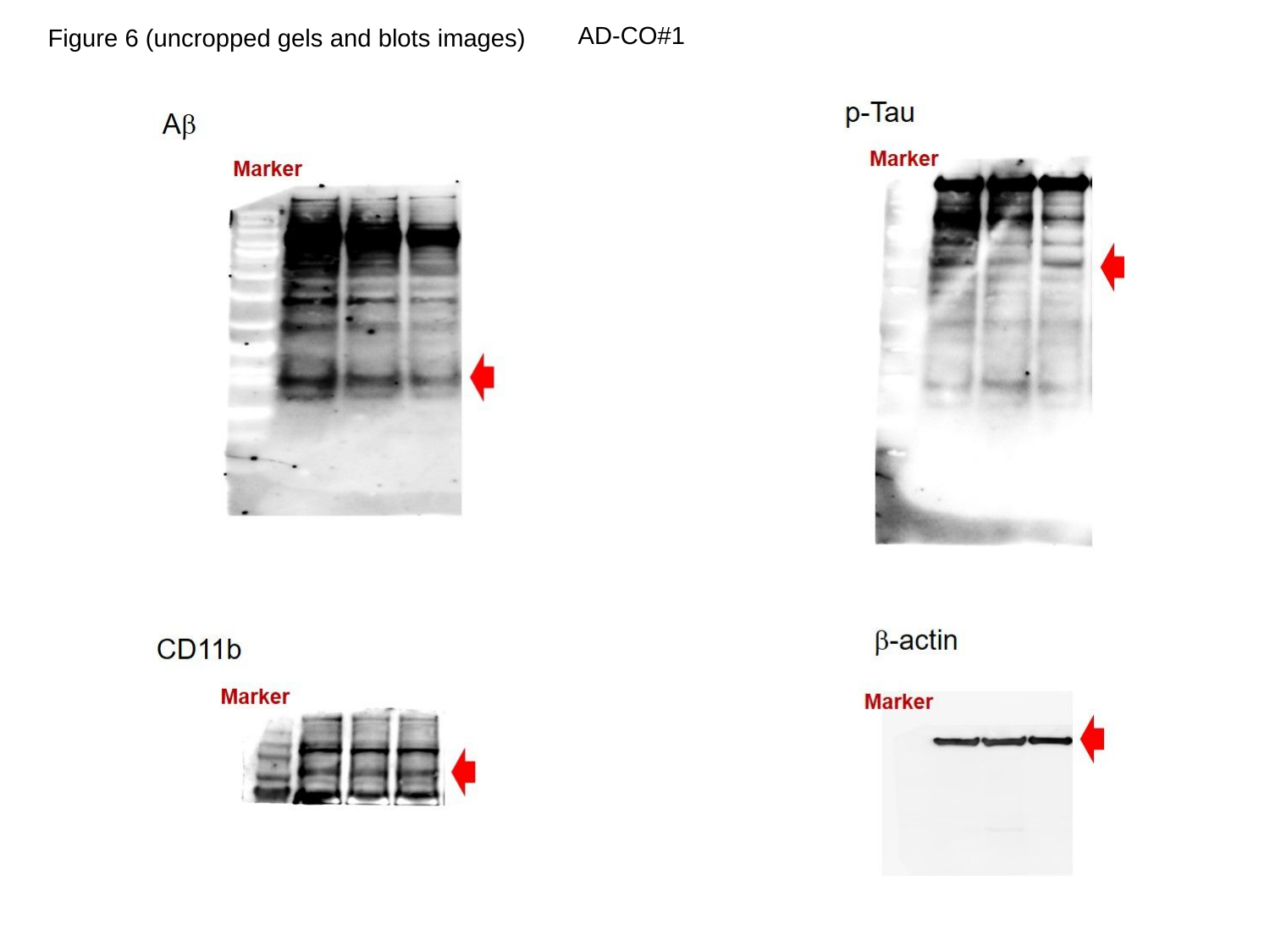

AD-CO#1
Figure 6 (uncropped gels and blots images)

## Slide 6
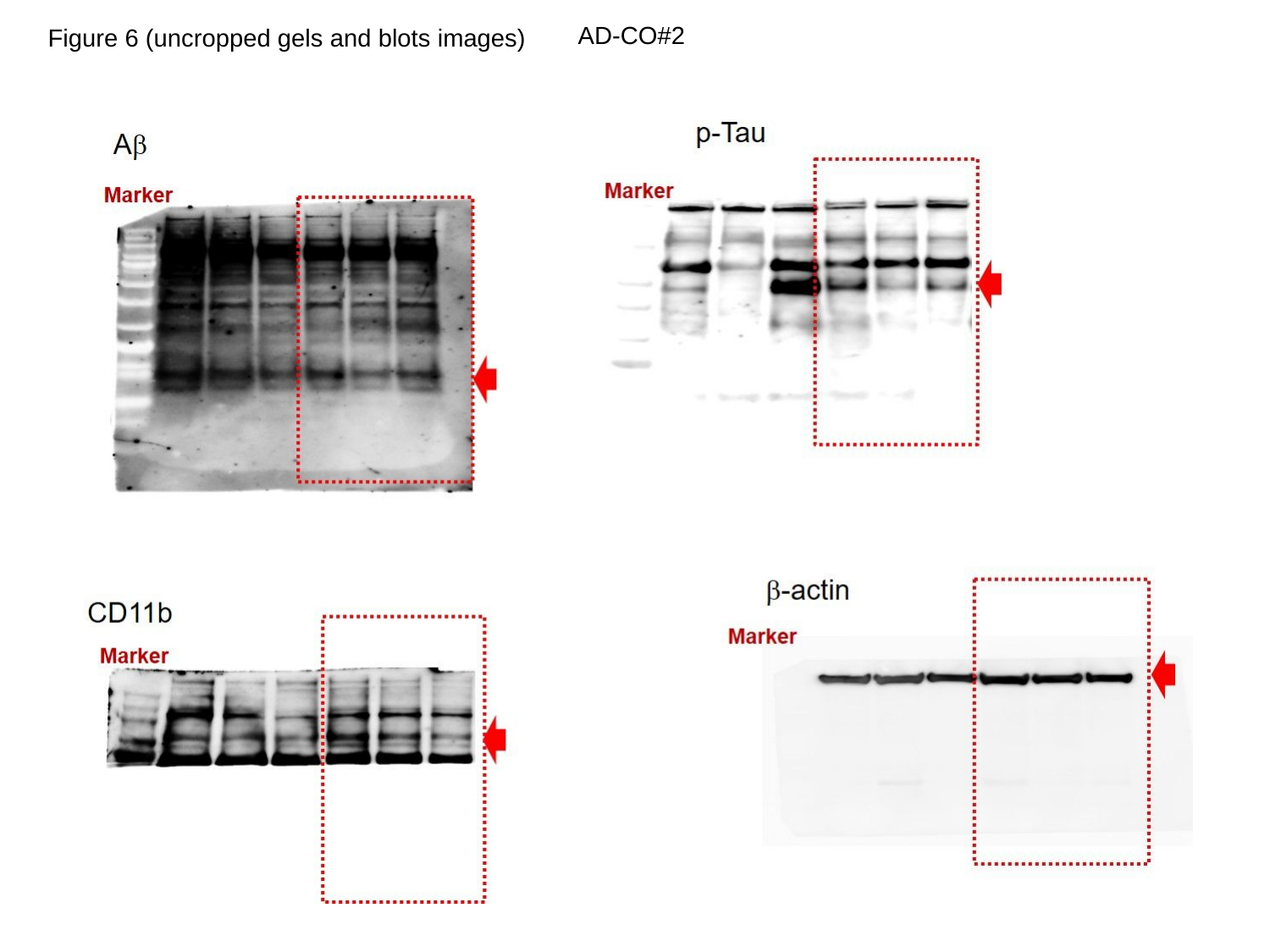

AD-CO#2
Figure 6 (uncropped gels and blots images)

## Slide 7
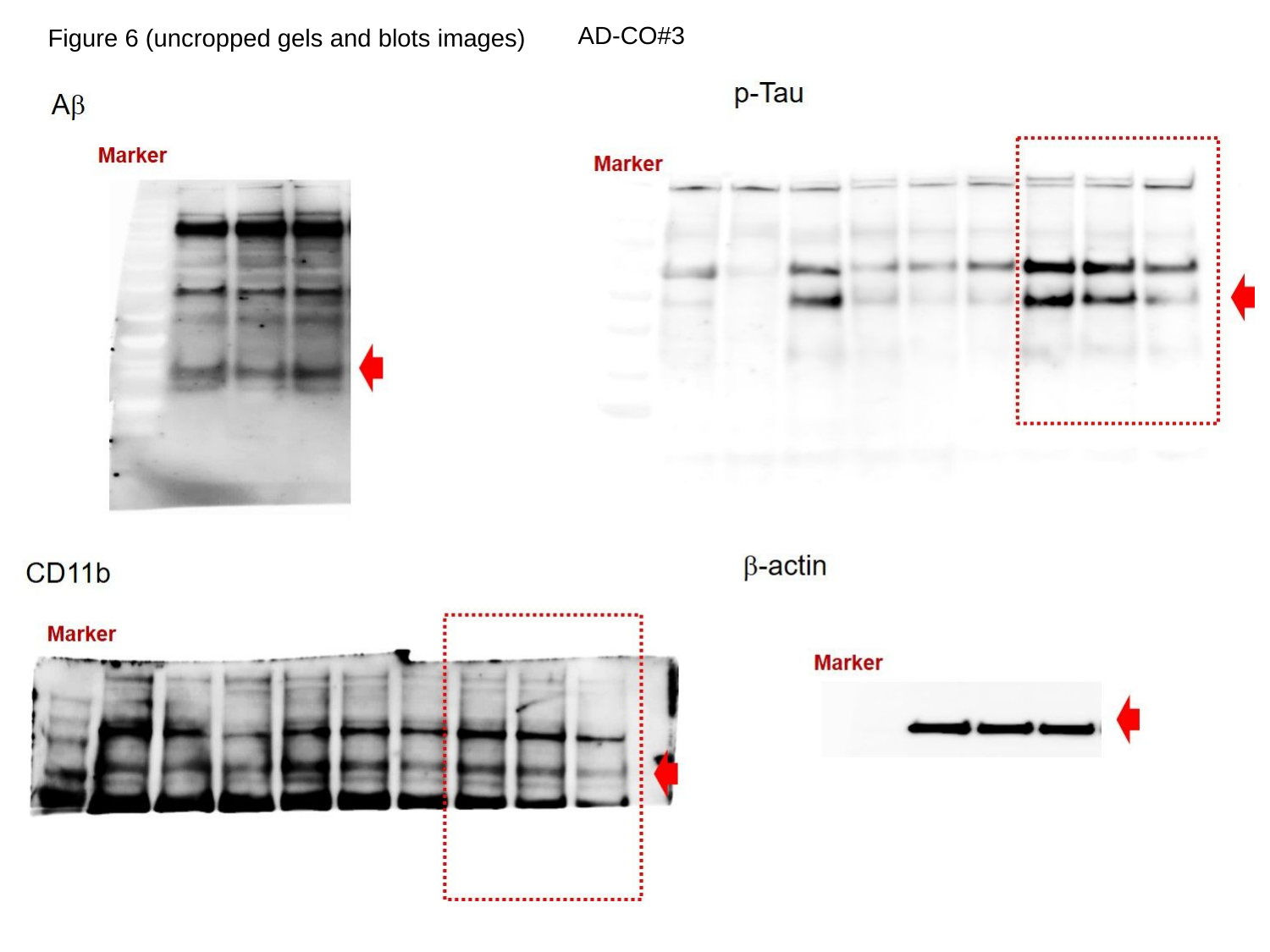

AD-CO#3
Figure 6 (uncropped gels and blots images)

## Slide 8
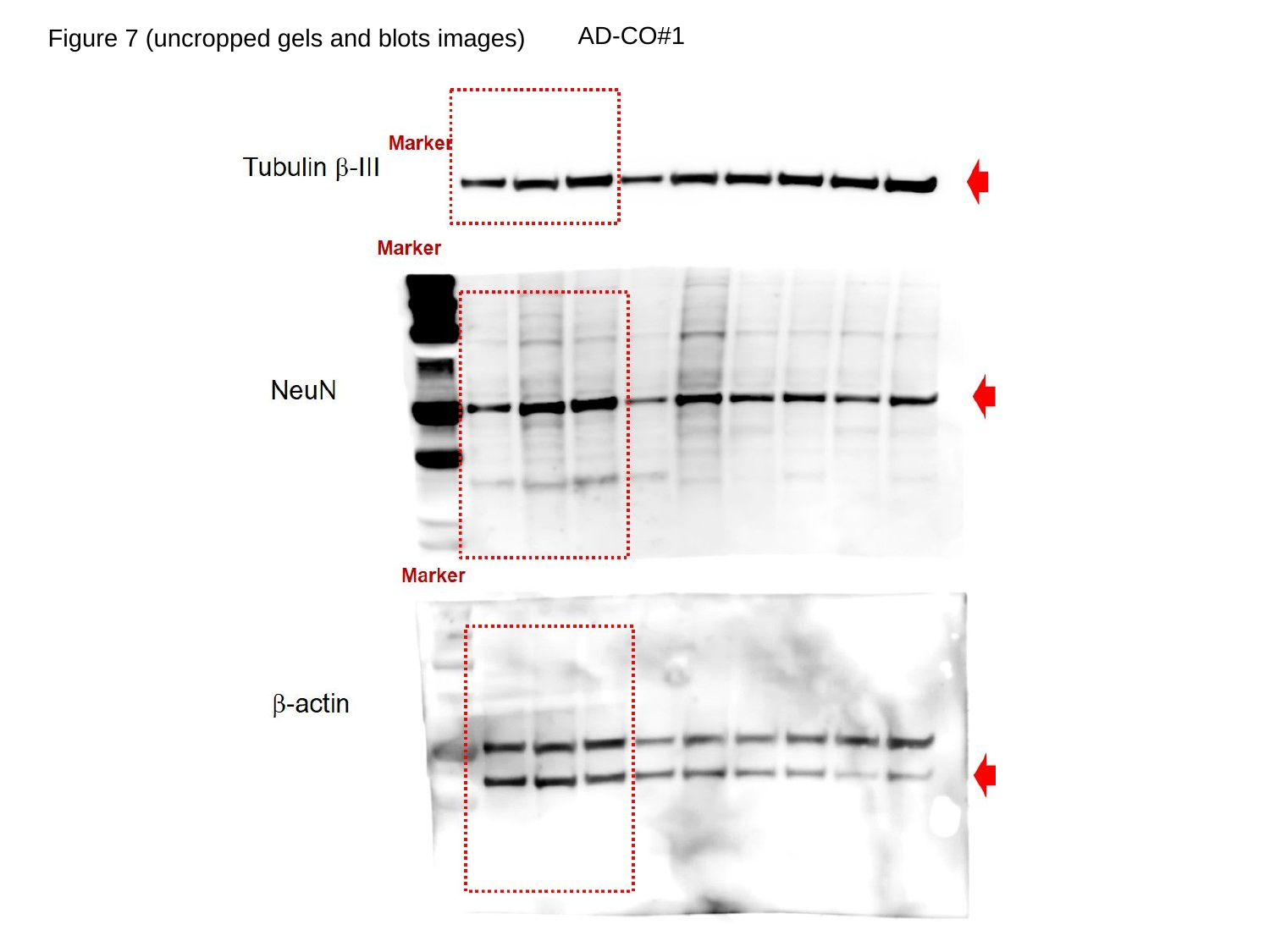

AD-CO#1
Figure 7 (uncropped gels and blots images)

## Slide 9
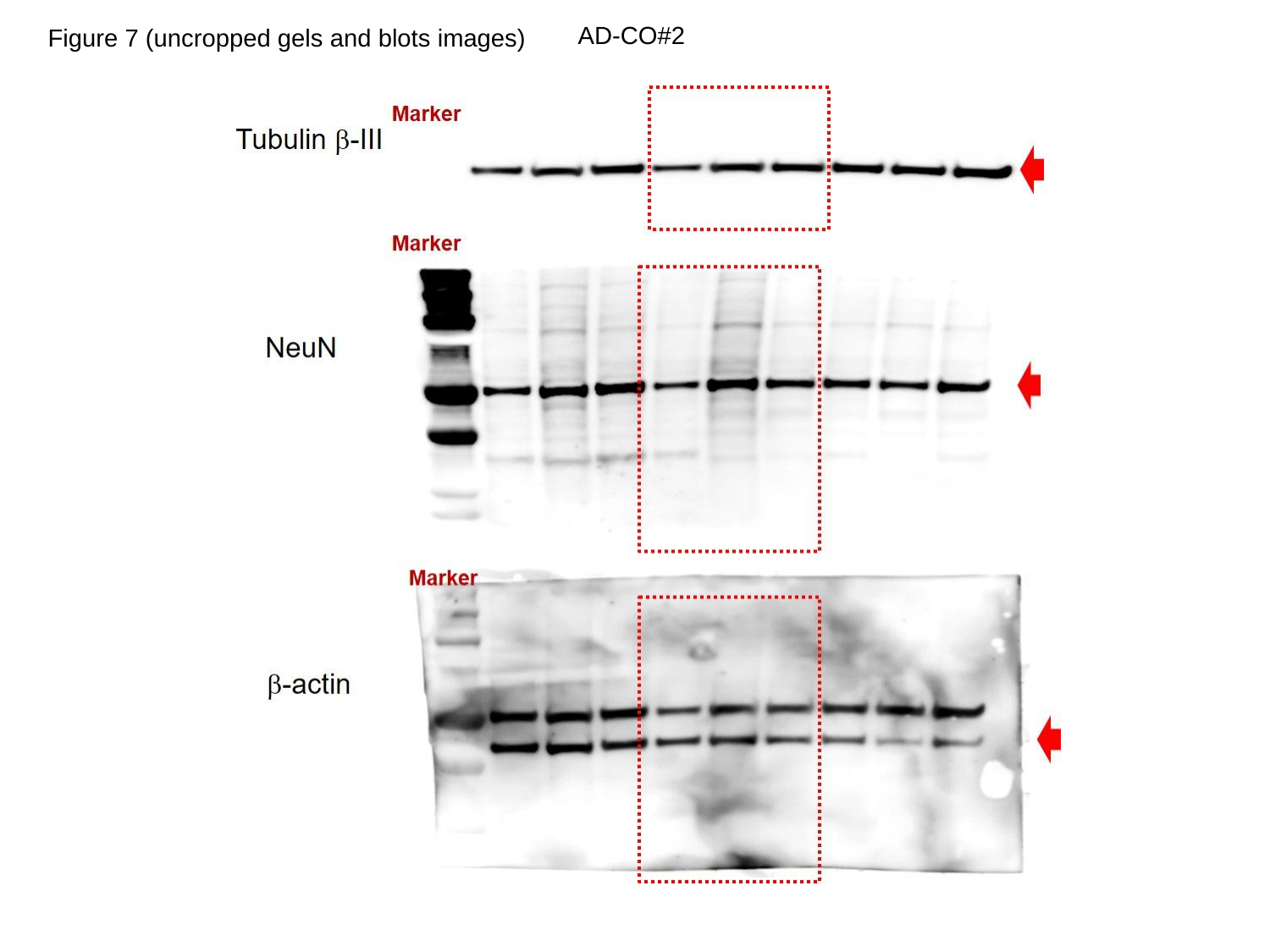

AD-CO#2
Figure 7 (uncropped gels and blots images)

## Slide 10
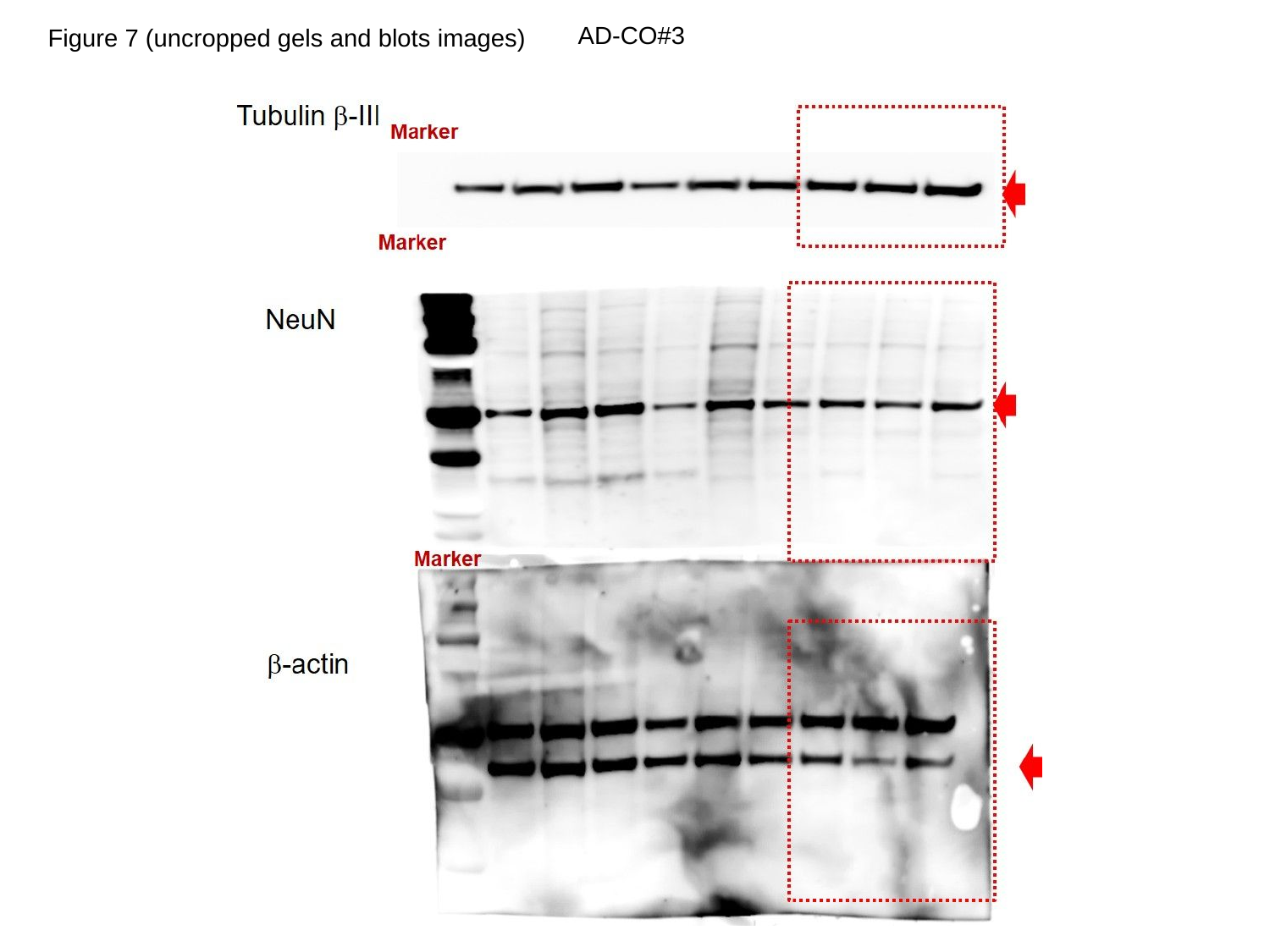

AD-CO#3
Figure 7 (uncropped gels and blots images)

## Slide 11
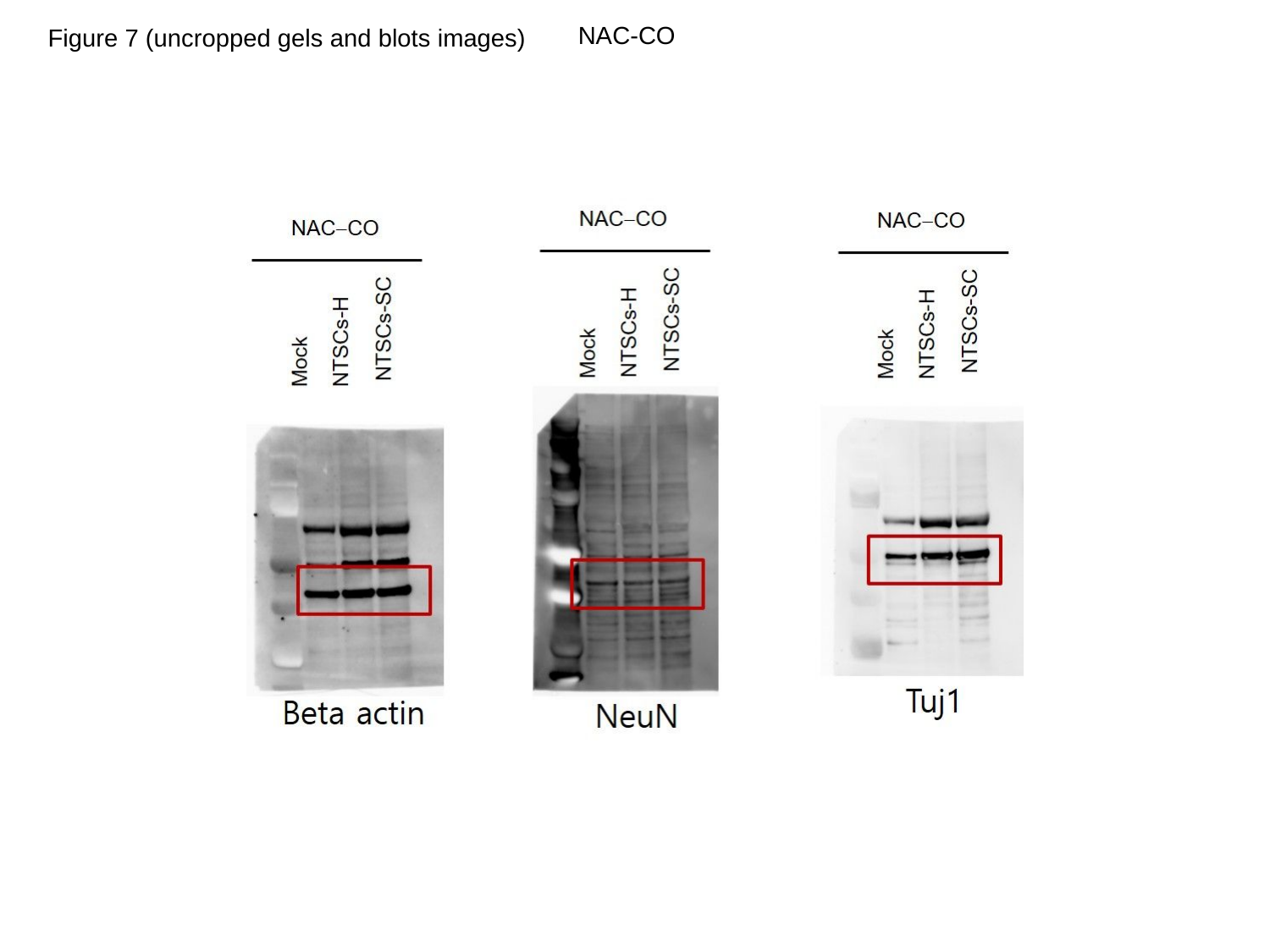

NAC-CO
Figure 7 (uncropped gels and blots images)

## Slide 12
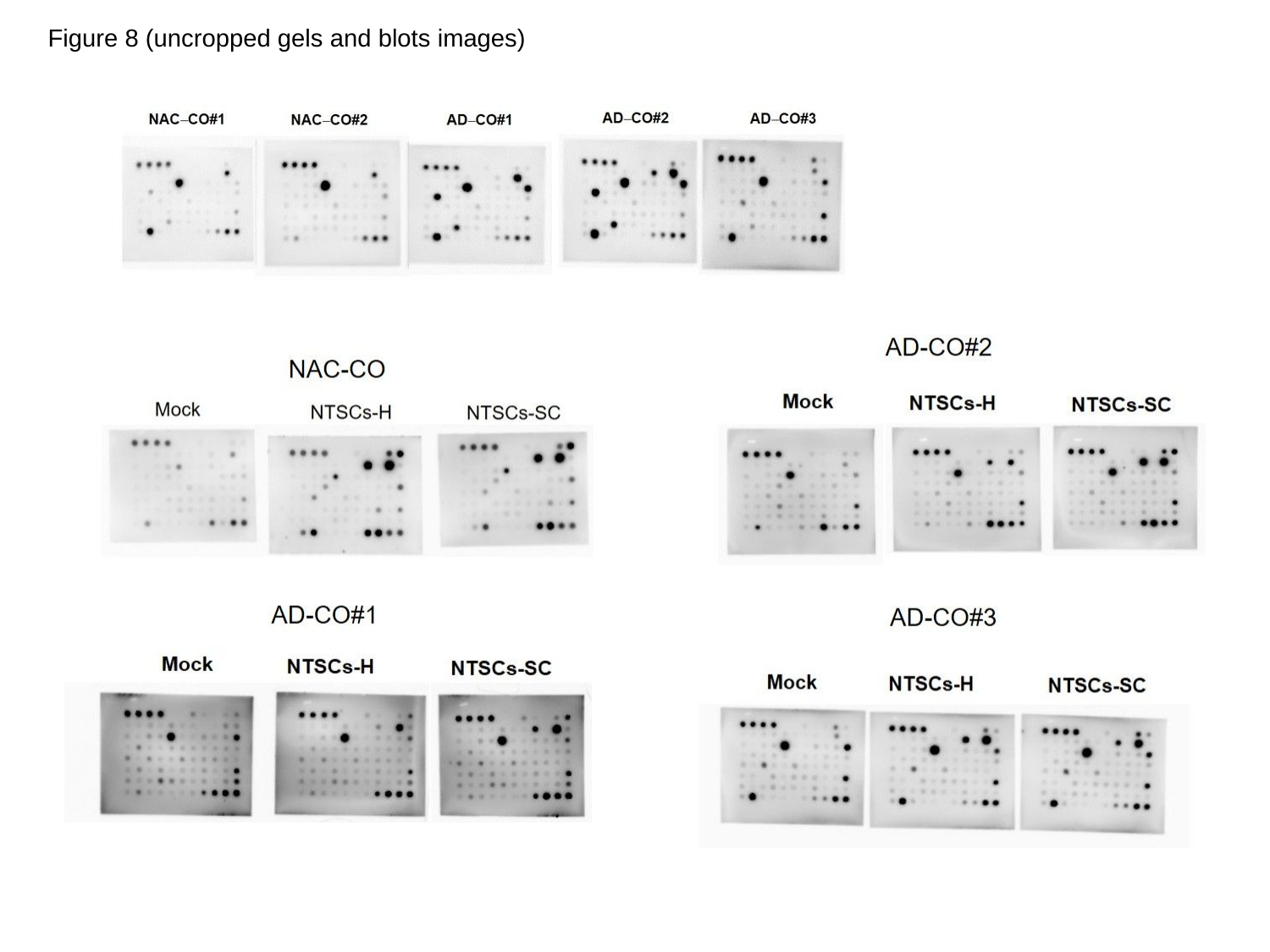

Figure 8 (uncropped gels and blots images)

## Slide 13
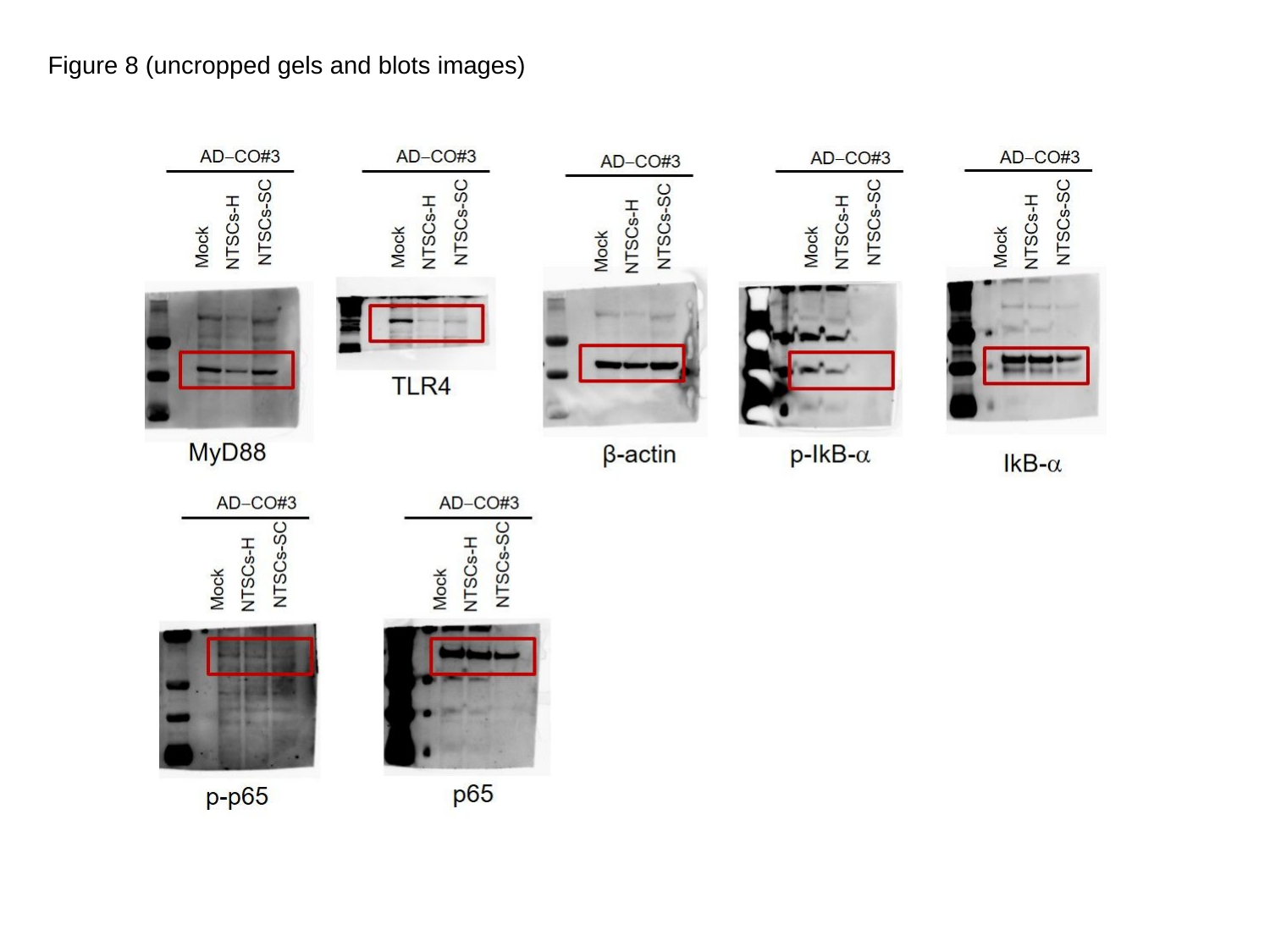

Figure 8 (uncropped gels and blots images)
